# Seafloor slopes control submarine canyon distribution: A global analysis

**DOI:** 10.1126/sciadv.adv3942

**Published:** 2025-06-11

**Authors:** Anne Bernhardt, Wolfgang Schwanghart

**Affiliations:** ^1^ Institute of Geological Sciences, Freie Universität Berlin, 12249 Berlin, Germany.; ^2^ Institute of Environmental Science and Geography, University of Potsdam, 14476 Potsdam, Germany.

## Abstract

Earth’s continental margins are dissected by submarine canyons that convey sediments, carbon, and nutrients to the deep ocean, regulating global biogeochemical fluxes. Despite their importance in the Earth system, the controls on canyon occurrence remain poorly understood. We report results from a spatial statistical model that explains global canyon distribution. By analyzing >2000 canyons, we show that canyon occurrence correlates with the inclination of continental slopes. Onshore orogeny and associated surface processes, long considered key controls on canyon formation, play a subordinate role. Instead, our results suggest slope inclination as the primary control on submarine canyon density. Because continental slope morphology is fundamentally shaped by marine tectonic and thermal processes, these large-scale forces indirectly govern canyon formation and distribution globally. As a result, they influence the presence of pathways that facilitate the transfer of sediments, carbon, and nutrients to the deep ocean, with implications for biogeochemical cycles over geological timescales.

## Teaser

Submarine canyon distribution is driven by slope inclination, linking tectonic and thermal forces to canyon formation.

## INTRODUCTION

Submarine canyons are deep, steep-sided valleys eroded into the seafloor along the world’s continental margins. They link terrestrial and deep-marine systems by transferring sediment, organic carbon, and pollutants (*1*–*3*). With lengths of up to 400 km and wall heights of up to 5 km, they dwarf their terrestrial counterparts (*3*). Similar to rivers on land, submarine canyons are key drivers in shaping the seafloor along Earth’s continental margins (*4*). Acting as conduits between land and the deep ocean, they embody what J. Syvitski termed the “global handshake between the coastline and the abyss.” Solid material transfer along the deeply incised canyons nourishes submarine fans with sediment including organic carbon, nutrients, and litter (*3*, *5*, *6*). Recent work has shown that submarine canyons play a vital role in the long-term global carbon cycle, as turbidity currents flowing along canyons bury around 62 to 90 million tonnes of terrestrial organic C/year, and these fluxes may have tripled during glacial times (*2*, *7*). Moreover, the abundance and position of submarine canyons with regard to terrestrial sources are crucial for terrestrial organic carbon capture and burial efficiency (*7*). In addition, submarine canyons influence ocean circulation and benthic biodiversity (*8*, *9*). Despite their widely recognized geomorphological and ecological importance, the factors controlling submarine canyon formation and distribution—whether they result from terrestrial dynamics and associated sediment dispersal or form independently of terrestrial processes—remain poorly understood.

**Fig. 1.**
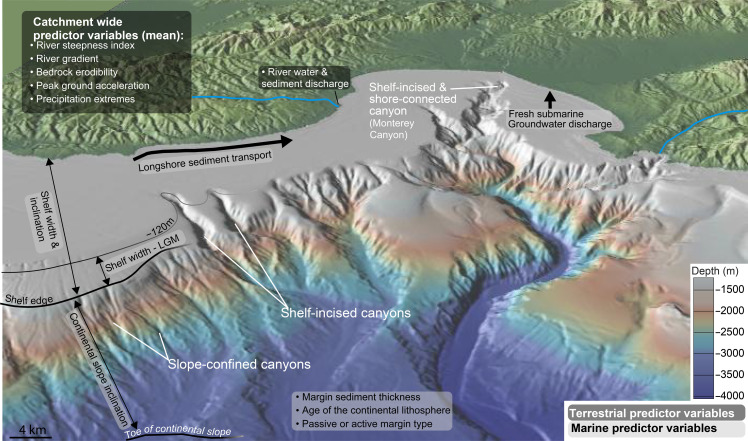
Submarine canyons and the potential controlling predictor variables of submarine canyon occurrence. Examples of different types of submarine canyons along the continental margin offshore Monterey Bay, California, featuring the prominent Monterey Canyon. Figure made with GeoMapApp (www.geomapapp.org) / CC BY (*84*). Terrestrial and marine predictor variables that potentially control submarine canyon formation are listed and explained.

To understand what controls submarine canyon occurrence, it is helpful to morphologically categorize submarine canyons into two types: slope-confined (or “blind”) canyons, which are exclusively located on the continental slope, and shelf-incised canyons, whose canyon heads eroded into the shelf (*10*, *11*). Shelf-incised canyons can be further classified as shore-connected if they extend to the coastline, where they link to terrestrial sediment sources, such as river outlets or longshore currents (Fig. 1). However, modern canyons that connect to the shore are rare—only about 4% of all canyon heads (and 11% of shelf-incised canyons) along the world’s major continental margins between 50°N and 50°S (*12*). Although some slope-confined canyons experience frequent turbidity current activity (*13*), studies show that shelf-incised canyons intercept river-derived and longshore sediment sources more effectively, making them particularly important conduits for transporting material to the deep ocean floor (*2*, *5*, *7*). In addition, they support more species-rich ecosystems compared to slope-confined canyons (*9*).

**Fig. 2.**
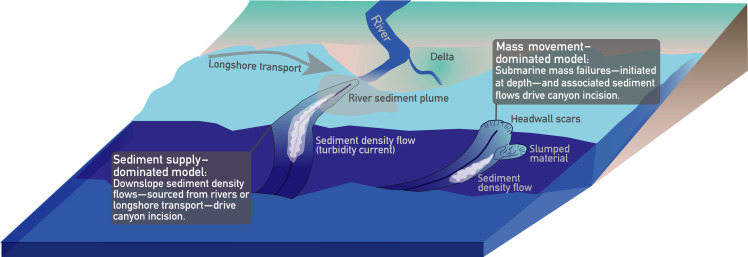
End-member models of submarine canyon formation driven by terrestrial sediment supply or mass movements. Sediment supply–dominated model: Canyon incision is initiated by erosive sediment density flows that are ultimately fed by terrestrially supplied sediment from rivers or longshore transport. Mass movement–dominated model: Canyon evolution is driven by internal mass failures along the continental slope and canyon flanks, with resulting erosive sediment density flows occurring independently of terrestrial sediment supply.

Two conceptual processes explain the formation of submarine canyons (Fig. 2): (i) The sediment supply–dominated model: This model emphasizes the role of sediment flows originating from upslope processes, particularly from continental sediment input. In this scenario, sediment supplied by rivers or longshore transport is ultimately transported downslope by sediment density flows, driving canyon incision from the shelf downward (often referred to as the “downslope erosion model”) (*14*–*16*). The erosion potential of these sediment flows is enhanced by high sediment supply and the presence of coarse-grained material derived from durable bedrock, which facilitates canyon incision and shaping (*17*). Notably, while downslope-directed sediment density flows drive erosion, they may result in headward incision and the formation of upstream-migrating knickpoints (*18*). (ii) The mass movement–dominated model: This model suggests that canyons originate from slope failures at deeper depths, with overall retrogressive erosion working upslope (often referred to the “upslope erosion model”) (*19*–*21*). The canyon formation process is thus dominantly driven by mass movements that gradually cut into the continental slope and shelf, capturing smaller gullies and expanding into complex canyon systems (*21*). Here, sediment flows are a consequence of mass failures and can further erode the canyon thalweg. Numerical modeling experiments highlight the crucial role of the critical slope stability in this mechanism (*22*). In summary, the central debate revolves around whether submarine canyons primarily develop through (i) sediment flows originating from terrestrially or longshore transport–derived sediment supply or through (ii) mass failures and associated sediment flows initiated at depth, with both processes likely contributing to canyon formation depending on the canyon type and environmental setting (Fig. 2).

So far, it remains unclear to what extent the processes conceptualized by the two end-member processes contribute to global submarine canyon distribution. Long-term observations of the ocean floor (e.g., multitemporal bathymetric data) documenting canyon evolution across different canyon types are largely unavailable due to the high logistical and financial costs associated with long-term repeated surveys in deep-sea environments. In addition, the timescales of canyon incision are likely very variable—ranging from decades to millions of years—making it difficult to design observation strategies that effectively capture the evolution of these systems. Numerical models that combine both upslope and downslope processes with tectonic uplift of the continental margin (*14*, *22*) show mixed results. A stratigraphic forward model suggests that most canyons form through landward retreat and gully capture, supporting the mass movement–dominated model (*21*). The modeling results are consistent with increased canyon occurrence along steeply inclined continental slopes (*8*, *19*). However, along the US West Coast, submarine canyons occur independently of shelf inclination or width. Instead, it has been proposed that their occurrence relates to durable lithologies in the bed load flux from terrestrial catchments (*17*). This suggests that offshore canyon formation is promoted in regions with onshore orogeny and related steep, ocean-draining river catchments that transport bed load sourced from resistant bedrock (*17*, *23*).

The contribution of these different processes to submarine canyon formation likely varies depending on their geographic and geological context. To decipher the major controls on canyon formation on a global scale, we apply spatial statistical techniques to predict submarine canyon distribution along the margins of major continents. Guided by previous regional and numerical modeling studies (*4*, *10*, *15*, *17*, *19*, *21*, *23*), we selected 16 terrestrial and marine explanatory variables (or “predictors”) that potentially predict spatial locations of submarine canyons or “canyon densities” along continental margins (Fig. 1 and table S1). If global submarine canyon formation is primarily driven by processes of the sediment supply–dominated model, then terrestrial factors—such as river steepness, seismic activity, riverine water and sediment fluxes, longshore sand transport, and shelf morphology—should correlate with canyon density. In addition, steeper shelves and continental slopes may facilitate faster and more erosive flows, potentially enhancing canyon incision and development. If the mass movement–dominated model applies, then higher canyon densities should occur in regions with steep continental slopes. If these slopes approach critical stability angles, then they will be more susceptible to sediment failure and submarine landsliding. Terrestrial processes should play a subordinate role, if any, when it comes to explaining the spatial distribution of canyons dominated by mass movements. Moreover, we propose that the controls on shelf-incised canyons transition from mass wasting–dominated to terrestrial sediment–driven processes as canyon heads approach areas influenced by shelf dynamics and terrestrial sediment input. We test these hypotheses with a global dataset of submarine canyons using point pattern analysis (*24*).

## RESULTS

### Global submarine canyon distribution

**Fig. 3.**
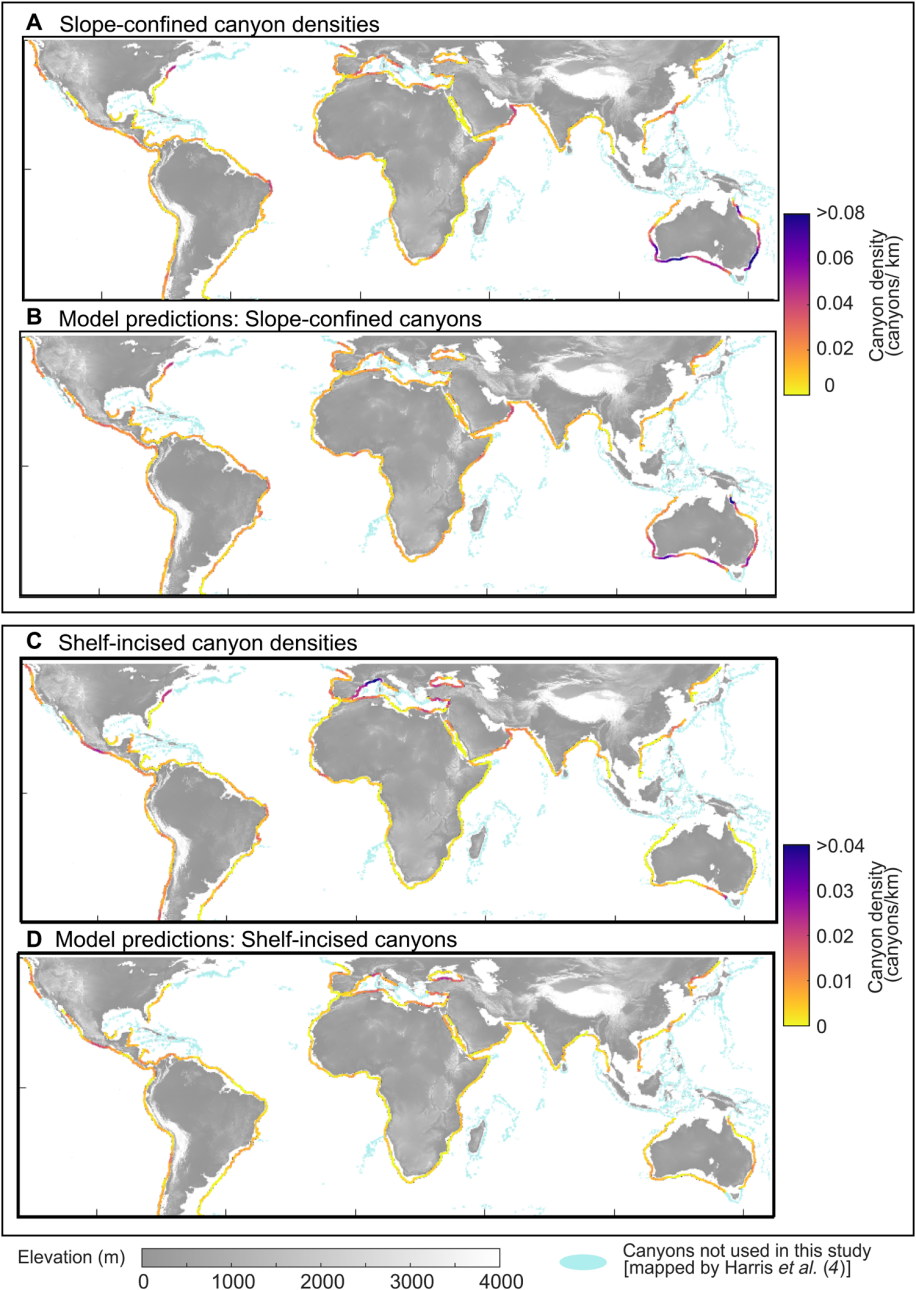
Global distribution of submarine canyons. (**A**) Original slope-confined canyon densities. (**B**) Model prediction of slope-confined canyon densities. (**C**) Original shelf-incised canyon densities. (**D**) Model prediction of shelf-incised canyon densities. Model prediction is based on point pattern statistics on a linear network using the frequentist predictive models.

To explore the global distribution of submarine canyons and the factors that influence their density, we analyze a spatial dataset of 2139 large canyons (water depth range, ≥1000 m; incision, ≥100 m) of which 605 are incised into the shelf (*4*). The canyons are distributed globally between 50°N and 50°S. We exclude islands, heavily glaciated margins, and regions with strong salt tectonism (e.g., Gulf of Mexico) (Fig. 3), as these areas are either not well suited to our inverse distance weighting approach (Materials and Methods) or are influenced by additional processes such as meltwater input and salt-related deformation. At the global scale, we conceptualize submarine canyons as points distributed along continental slopes by locating the centroid of each mapped canyon polygon and projecting them onto the centerline of the slope (fig. S1). Our data thus comprise a set of points attached to a network of lines and are consequently treated as a point pattern on a linear network (*25*, *26*). The centerline of the continental slope has a length of 104,200 km, so that canyons occur at a density of ~20 canyons per 1000 km on a global average. By conceptualizing canyons as points, we focus on their distribution relative to the slope centerline. This simplification reduces storage and computational requirements, thus enabling us to develop spatial statistical models of their global distribution and the factors that control it.

### Predictor variables and statistical models

To assess the variables influencing submarine canyon density, we compiled 16 candidate predictors based on previous studies (Fig. 1 and table S1) (*4*, *27*–*36*). These predictors encompass terrestrial and marine variables to account for the diverse processes potentially influencing submarine canyon formation. First, we classified continental margin type as a categorical variable, distinguishing passive from active margins. Among the terrestrial variables, we included the mean gradient of onshore catchments, the mean river steepness index, the mean annual discharge, and the modeled suspended sediment flux before substantial human impact—all of which represent onshore topography, sediment supply, and fluvial dynamics. In addition, we considered peak ground acceleration (for a 10% exceedance probability within 50 years) as a proxy for seismic activity, the weighted global erodibility index to reflect landscape susceptibility to erosion, and the ratio of the 90th to 50th percentile of mean annual rainfall to capture variability in precipitation (Fig. 1 and table S1). The marine variables include the inclination of the adjacent shelf and continental slope, the mean shelf width under present-day and Last Glacial Maximum (LGM) conditions (when sea level was ~120 m lower than today), the modeled fresh submarine groundwater discharge, the sediment thickness along the continental margin, and the age of the continental lithosphere. In addition, we incorporated longshore wave–induced sand transport to account for coastal sediment redistribution (Fig. 1 and table S1). Table S1 presents all predictor variables, their data sources, data processing, and the reasoning behind their inclusion in more detail. These explanatory variables ideally account for processes of incipient canyon formation and evolution. Some variables required preprocessing (table S1). For example, to avoid canyon topography influencing slope inclination, we created a smoothed surface extending from the shelf edge to the toe of the slope, resulting in the slope inclination for the undissected continental slope. The same approach was used to compute regional shelf width and inclination. This ensures that calculated slope inclinations are not affected by the presence of canyons, preventing circular reasoning in the model where canyon density would otherwise be implicitly embedded in the predictor variable. This global-scale approach, which relies on spatially averaged regional slope inclinations rather than local measurements, entails some smoothing of local topographic features [e.g., growing anticlines or faults that can locally oversteepen canyon walls, leading to failure (*37*)] and of higher slope inclinations near canyon heads and walls—critical areas where flows may be triggered and potentially influence canyon initiation and development.

We evaluated the variables’ ability to predict canyon locations using an inhomogeneous Poisson point process model where the intensity function is a log-linear model of the predictor variables. To identify and select variables supporting either the sediment supply–dominated or the mass movement–dominated model, we applied both a frequentist approach (stepwise regression) and a Bayesian approach (Bayesian penalized regression). We fit separate models for slope-confined and shelf-incised canyons to evaluate whether formation processes and their controls vary between the two canyon types.

### The dependence of canyon density on bathymetric resolution

**Fig. 4.**
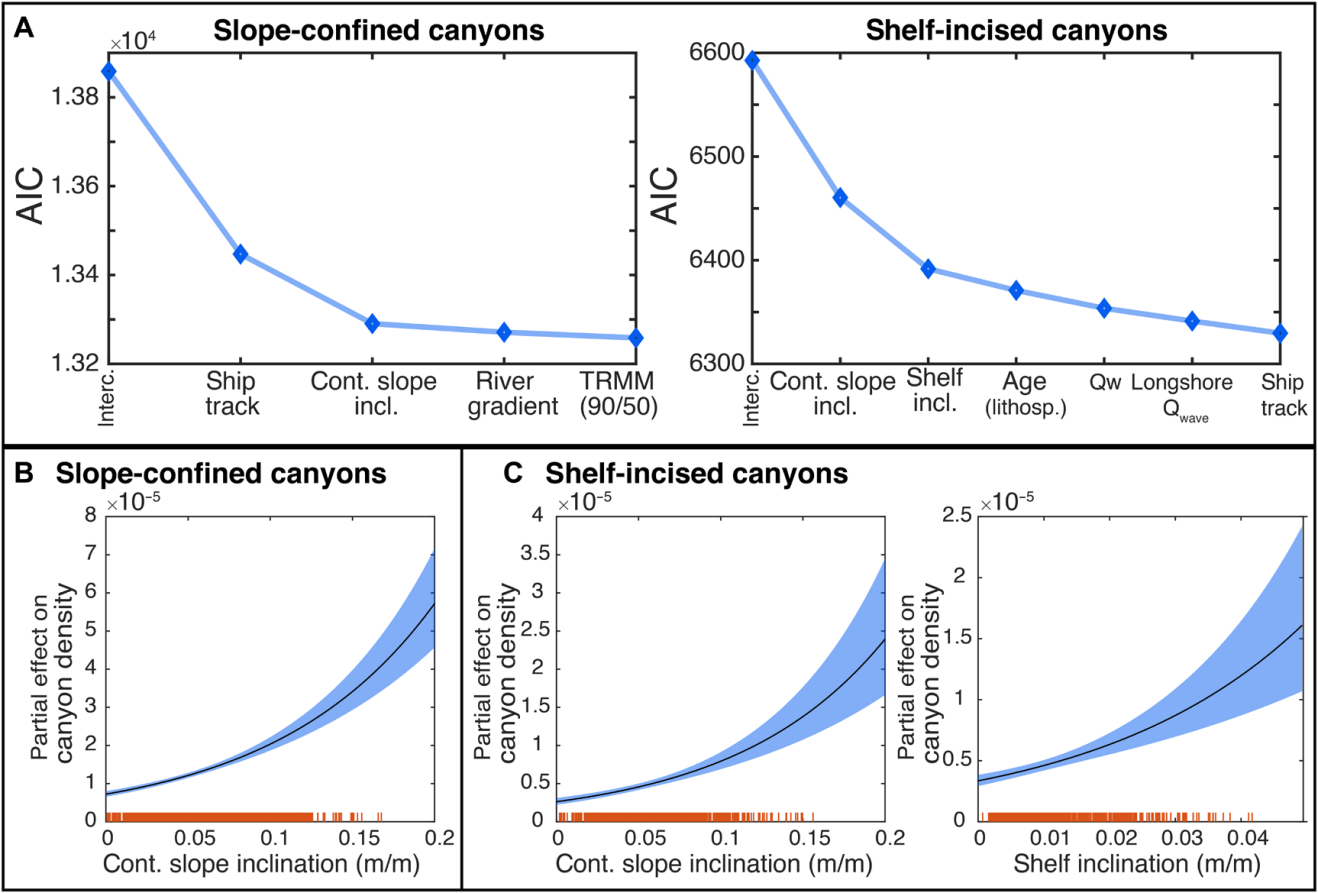
Results of the inhomogeneous Poisson point process model. (**A**) Development of the AIC for the frequentist inhomogeneous Poisson point process model as explanatory variables are added. The AIC is a measure used to compare statistical models by balancing goodness of fit and complexity—a lower AIC indicates improved model performance. Note that the improvement in model performance is indicated by the reduction in AIC when a specific variable is added to the model. Predictor variables are presented in order of the largest reduction in AIC, and only predictors that introduce the highest AIC reductions are shown. Ship-track density is included as a predictor to account for data quality variations but does not represent a physical process driving canyon formation. (**B** and **C**) Partial effects of single predictor variables in the model. (B) The partial effect of continental slope inclination on slope-confined canyon density and (C) the partial effect of continental slope and shelf inclination on shelf-incised canyon density. These plots show how the canyon density varies when this variable is changed, while all other variables are kept constant. Orange lines indicate canyon (point) locations along the slope centerline network. Cont. slope incl., continental slope inclination; shelf incl., shelf inclination; Qw, weighted mean annual water discharge of onshore catchments; TRMM (90/50), ratio of the 90th to 50th percentile of mean annual rainfall; Q_wave_, longshore wave–induced sand transport; age (lithosp.), age of underlying oceanic lithosphere; ship track, ship-track density (see table S1 for a detailed explanation of predictors); interc., intercept only. This means that no predictor variables are included in the model—only the intercept term is used. This results in a baseline model where the predicted outcome is simply the average of the response variable, without accounting for any predictors.

To better understand the controls on submarine canyon distribution, we analyzed previously mapped submarine canyons delineated from a global bathymetric model based on satellite gravity data calibrated with single-beam and multibeam sonar soundings (*4*). As the resolution of this model—and thus the accuracy of mapped canyon density—depends on the density of calibration soundings (*5*, *12*), we included ship-track density as an additional variable to account for differences in data quality. This global bathymetric model is based on satellite gravity data, and the gravity-to-topography ratio is calibrated with soundings from single-beam and multibeam sonar data. A high sounding density improves the resolution of seafloor details (*27*, *38*), and we find that ship-track density (from which soundings were used for calibration) exerts a primary control on the spatial density of slope-confined canyons (Fig. 4A and figs. S2 and S3). In the subsequent analysis, we thus added ship-track density as an additional explanatory variable to account for variable data quality. The area under the curve (AUC) metrics of models with ship-track density alone are 0.65 for slope-confined and 0.60 for shelf-incised canyons (AUC = 1 represents a perfect classifier). A Kolmogorov-Smirnov test reveals that the remaining variability of global canyon densities cannot be explained by a completely spatially random model, suggesting that there are further controls on canyon density (Materials and Methods).

### Important predictor variables

To better understand the variables influencing submarine canyon distribution, we analyzed the relationship between canyon densities, 16 predictor variables, and ship-track density. We identified the most influential variables using model evaluation metrics, such as the Akaike Information Criterion (AIC), to quantify each variable’s contribution to model performance. Our results show that the global occurrence of both canyon types is primarily related to regional continental slope inclination, alongside ship-track density as a proxy for data quality (Fig. 4A, figs. S2 and S3, and tables S2 and S3). The AIC assesses model performance by balancing goodness of fit and model complexity, with lower values indicating better models (Fig. 4A and fig. S3). A drop in AIC when adding a variable signifies improved explanatory power, while a minimal change suggests limited contribution. Incorporating continental slope inclination into the model yields the highest reduction in AIC for both canyon types (AIC, −157 for slope-confined canyons; AIC, −132 for shelf-incised canyons) (Fig. 4A) and is positively related to canyon densities (Fig. 4, B and C). In addition to continental slope inclination, the density of shelf-incised canyons increases with higher steepness of the adjacent shelf (AIC, −69). All other variables reduce the AIC for <20 and are not considered further here, as they do not enhance the model performance significantly.

For both canyon types, densities double if continental slope inclination increases from 0.05 to 0.15 m/m (2.9° to 8.5°) (Fig. 4B). Regional, smoothed slope inclinations up to 0.05 are recorded for 77% of the world’s continental slopes (e.g., Atlantic passive margin offshore Namibia, South Africa, and Argentina), whereas 23% have inclinations between 0.05 and 0.15 m/m (e.g., Pacific coast of Central and South America, Mediterranean margin offshore Algeria and the Ligurian Sea, and Great Barrier Platform off northeastern Australia). Moreover, the density of shelf-incised canyons doubles if shelf inclination increases from 0.01 to 0.03 m/m (0.6° to 1.7°) (Fig. 4C). Shelf inclinations of up to 0.01 are recorded for 78% of the world’s continental slopes (e.g., Atlantic passive margin and Australia), whereas 20% have shelf inclinations between 0.01 and 0.03 m/m (e.g., Pacific coast off northern Chile and Mediterranean margin offshore Algeria and Liguria). There is no minimum threshold in slope and shelf inclination below which canyons do not occur.

### Model residuals and canyon clustering

**Fig. 5.**
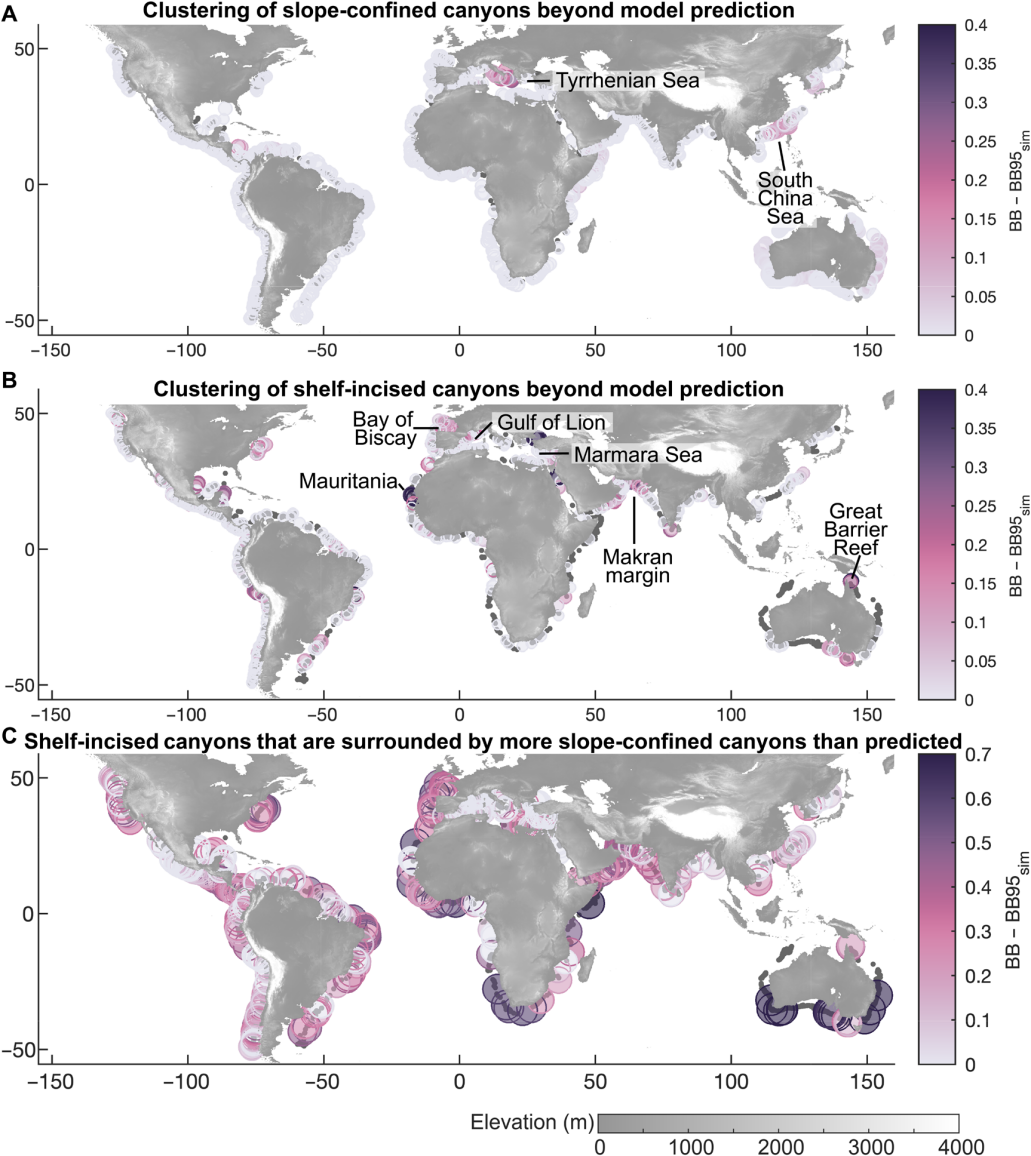
Remaining canyon clustering (not predicted by the model). (**A** and **B**) To illustrate the residual canyon clustering or hotspots, we compare the actual join count [black-black (BB)] to the simulated 95th percentile (BB95sim_canyon_) by subtracting $\text{BB}-\text{BB}95_{\text{sim}}$ . A higher $\text{BB}-\text{BB}95_{\text{sim}}$ value indicates stronger clustering. The bubble color represents this difference for (A) slope-confined canyons and (B) shelf-incised canyons. (**C**) To assess whether shelf-incised canyons are surrounded by more slope-confined canyons than explained by the Bayesian model, we analogously compare the actual join count to the simulated 95 percentiles. Gray-filled dots indicate the center points of shelf-incised canyons in (A) and slope-confined canyons in (B) and (C).

Inhomogeneous Poisson point process models account for first-order effects of external variables on canyon densities but do not consider interactions between canyons. These second-order effects imply that the presence of a canyon affects the probability of canyon occurrence nearby. To account for canyon interactions, we used operational join counts, a spatial statistic that quantifies the co-occurrence of categorical variables in neighboring locations. In this specific case, we measure whether observed canyon types occur in spatial patterns predicted by our model or appear clustered or dispersed beyond the model prediction (Fig. 5 and figs. S4 to S6) (*39*). Specifically, the operational join count measures residual canyon clustering by assessing whether a canyon of a given type is surrounded by more canyons of the same or the other type than predicted by the model, with significant deviations indicating spatial interactions not accounted for by the model. To test for significant clustering, we simulated 1000 join counts under the assumption that canyons follow the modeled inhomogeneous Poisson point process and calculated the 95th percentiles of these simulations (Materials and Methods). If the observed join count exceeds this threshold, then it indicates canyon clustering beyond first-order effects, revealing canyon interactions not captured by the model. In particular, we assessed whether slope-confined or shelf-incised canyons appear in these clusters (Fig. 5, A and B) or whether shelf-incised canyons are surrounded by more slope-confined canyons than expected (Fig. 5C).

Our results indicate that shelf-incised canyons are surrounded by more slope-confined canyons than predicted along almost all continental margins (Fig. 5C). Exceptions include margins with very high shelf-incised canyon densities such as the western and eastern Mediterranean and the west coast of Central America. We interpret these results to reflect a positive feedback in the formation of shelf-incised canyons. Their growth leads to an advantageous position in competing for longshore currents, groundwater (*40*), and erosive sediment sources, inhibiting the growth of nearby slope-confined canyons. Previous analysis of the width-depth ratios of canyons has also highlighted the importance of these interactions (*41*).

## DISCUSSION

### Controls on global submarine canyon distribution

Canyon densities obtained from random realizations of our point process model are generally consistent with observed global densities, with particularly good agreement for slope-confined canyons. While the model also captures the distribution of shelf-incised canyons to some extent, its predictive skill is notably stronger for slope-confined systems (Fig. 3 and fig. S6). The most important predictor variables are continental slope inclination, followed by shelf inclination, which is relevant only for shelf-incised canyons (Fig. 4). Steep continental slopes can occur along passive and active margins, but maximum values are reached along active margins (fig. S7). However, shelf inclinations are remarkably higher along active margins when compared to passive margins (fig. S7). Going a step further, first-order controls on continental slope inclination include margin uplift, basin-floor subsidence, tectonic deformation, margin formation by sedimentation over geologic timescales, and the composition and texture of the slope-constructing sediment and rock (*42*). The steeper shelf inclinations along active margins are due to uplift from the build-up of accretionary sediments, folding and faulting, and magmatic intrusion and extrusion (*43*). Along passive margins, steep continental slopes are related to high thermal seafloor subsidence (*43*) or low long-term sediment supply (*42*, *44*). Moreover, rock properties control the geometry of continental margins; for example, carbonate margins are steeper due to greater shear strength (*45*), while muddy, cohesion-dominated sediment forms gentler slopes (*44*). In turn, long-term sediment supply and nature of this sediment should alter the inclination of the continental slope to different degrees. However, margin sediment thickness—a marine variable approximating sediment supply over geologic timescales—does not correlate with shelf and slope inclination (fig. S7) and is largely irrelevant when predicting canyon density (Fig. 4 and tables S2 and S3). While long-term sediment supply may influence slope inclination to some extent, tectonic and thermal processes play a dominant role in shaping continental margins, ultimately setting the stage for canyon formation. Steep margins are generally more prone to mass failure and retrogressive erosion (*46*, *47*) [except where low sedimentation rates and frequent earthquakes lead to slope stabilization (*48*)], which can result into incipient canyon formation. Hence, submarine canyon distribution is ultimately controlled by the tectonic regime, margin cooling, and the physical properties of the margin-building material.

Past sea level fluctuations during glacial-interglacial timescales can directly influence canyon connectivity to terrestrial sediment sources and potentially affect submarine canyon formation. During periods of low sea level, canyon systems often experience increased sediment transport, as sediment input from rivers and longshore transport increases (*5*). Previous work has assessed the proximity of canyon heads to the −120-m elevation contour (*12*, *49*), which approximates the shoreline during the LGM when the sea level was around 120 m lower than today (*50*). Reanalysis of these data reveals that ~79% of canyons of this study remained disconnected from the shore even under these extreme low–sea level conditions (fig. S8). Specifically, 96% of slope-confined canyon heads remain disconnected during the LGM, whereas nearly 50% of shelf-incised canyons were located within 6 km of the LGM shoreline and can thus be considered connected to the shore. Consequently, while glacio-eustatic sea level fluctuations likely influenced the formation of many shelf-incised canyons, only a small proportion of slope-confined canyons analyzed here was directly connected to rivers or longshore sediment transport during these lowstands. This may also account for the significantly better model performance observed for slope-confined canyons compared to shelf-incised ones. Our findings therefore suggest that most submarine canyons—particularly slope-confined canyons—are primarily shaped by slope-driven processes and margin dynamics, rather than by direct river connectivity, even under conditions of extreme sea level lowstands.

Building on these findings, our analysis suggests that the global distribution of both submarine canyon types is largely consistent with the mass movement–dominated model and that the formation of slope-confined canyons remains largely unaffected by terrestrial processes. As opposed to previous studies (*10*, *17*, *23*), terrestrial parameters related to sediment supply—such as onshore tectonic activity, relief, river water and sediment discharge, river steepness, and onshore bedrock erodibility—play a subordinate role in the prediction of submarine canyons between 50°N and 50°S. However, steep continental slopes do not exclusively favor the mass movement–dominated model. In the framework of the sediment supply–dominated model, steep slopes can also enhance erosion as sediment density flows accelerate and become more erosive on steep slopes, contributing to canyon formation. However, the fact that slope inclination is the only significant parameter in predicting slope-confined canyons further supports the dominance of the mass transport–dominated model. In contrast, the positive correlation between shelf-incised canyon density and increasing shelf inclination suggests that shelf processes play a substantial role in canyon head incision. Steeper shelf inclinations can enhance across-shelf suspended sediment transport (*51*), and we therefore infer from this correlation an increased influence of downslope erosional processes driven by sediment density flows—ultimately sustained by terrestrial sediment supply (*15*). Our analysis shows that shelf and continental slope inclinations are only very weakly correlated (coefficient of determination = 0.17) (fig. S7). Therefore, these parameters can be regarded as largely independent controls in submarine canyon formation.

Ultimately, the density of submarine canyons along continental margins is primarily controlled by regional seafloor topography, which defines the potential—but not the magnitude—for land–to–deep-sea sediment transfer. While a high canyon density increases the capacity for material transport, actual sediment flux is highly variable and strongly governed by factors such as fluvial sediment discharge, longshore sediment supply, and the degree of connectivity of these sources to canyon heads. Although shelf-incising canyons represent only a subset of the global canyon population, they likely contribute disproportionately to sediment and carbon transfer, particularly when connected to major river systems such as the Bengal or Congo canyons (*2*, *7*). Our reanalysis indicates that approximately half of all shelf-incising canyons were likely connected to rivers or littoral cells during the LGM, when the sea level was ~120 m lower than the present. These canyons, which can act as active conduits under lowstand conditions, therefore represent critical pathways to deep-sea sediment and carbon fluxes, despite being less numerous than slope-confined canyons.

As previously noted, continental slope inclination is, by far, the most important predictor variable, with steeper slopes strongly associated with increased canyon densities, supporting the mass movement–dominated model. Hence, river outlets and submarine canyon heads first approach one another from above and below the coastline without immediate or quantifiable interaction. Nevertheless, previous studies have shown that while terrestrial surface processes generally play a subordinate role in initial submarine canyon evolution, they become increasingly important when maintaining direct connections of coastal sediment sources and canyon heads (*12*). This earlier study explored the key controls on canyon head connectivity to the shoreline using a similar set of explanatory variables but applied Bayesian regression modeling instead of a point pattern analysis along a linear network–based approach. This study, along with other prior research, demonstrated that canyon head connectivity is governed by factors such as shelf inclination and morphology, terrestrial bedrock erodibility, and water discharge (*12*, *23*). Once a canyon head intersects a terrestrial sediment source, the sediment routing system efficiently transfers sediment and organic carbon to the deep sea, with the characteristics of the terrestrial sediment source directly influencing canyon head positioning.

### Limitations and unaccounted variables in canyon density predictions

Despite the general agreement between predicted and observed canyon densities along continental margins, notable discrepancies remain—especially in the prediction of shelf-incised canyons (Fig. 3 and fig S6). To quantify model residuals, we applied operational join-count statistics (Fig. 5, A and B, and figs. S4 and S5) (*39*). Our results indicate that slope-confined canyons largely follow the distribution expected under the null hypothesis, which, in our case, refers to the predicted densities obtained from the point pattern model. In contrast, shelf-incised canyons form clusters that exceed model expectations in regions such as the South China Sea, Tyrrhenian Sea, Makran margin, Bay of Biscay, Gulf of Lion, offshore Mauritania, northern Marmara Sea, and the Great Barrier Reef (Fig. 5, A and B). These differences can arise primarily from two factors: the influence of additional controlling variables not included in our models (*52*) and interactions between canyons.

The clustering of canyons not predicted by the model may stem from missing predictor variables that are unavailable at the scale required for our study (Fig. 5). First, underlying tectonic structures can promote canyon formation, but their location is often poorly known. Faults, for instance, can create zones of lithological weakness, as shown for canyons in western and eastern Australia, where they align with ancient structures formed during the opening of adjacent ocean basins (*8*). Similar relationships are observed in the heavily fault-dissected Tyrrhenian Sea (*53*) and Marmara Sea (*54*), where torrential outflows of Black Sea water during meltwater phases contributed to canyon formation. Along convergent margins, such as the Makran margin, shelf-incised canyons are more abundant than predicted, likely due to enhanced erosion driven by knickpoint retreat along thrust faults and oversteepened deformation fronts (*55*). Second, the physical properties and associated erodibility of shelf- and slope-forming material influence canyon abundance but remain poorly quantified at the global scale. For example, canyon clustering along the reef front of the northern Great Barrier Reef (Australia) suggests that carbonate dissolution and initial forereef morphology modulate canyon formation. In contrast, the carbonate front of the Blake Plateau (the Blake escarpment) along Florida’s Atlantic coast shows minimal canyon formation (Figs. 3 and 5) (*4*, *56*), likely due to the low erodibility of its steep platform front. In addition, the position of shelf-incised fluvial valleys and older buried submarine canyons (*11*) serves as structural weak zones that facilitate canyon erosion, but their distribution remains largely unknown on a global scale. Third, erosive density currents can be triggered by “dense shelf-water cascades” (*57*). This phenomenon arises from differences in water density caused by variations in temperature and salinity of shelf waters, resulting in descending offshore flows. These currents erode canyon heads and floors and are observed in regions such as the Gulf of Lions (*58*) and offshore southeastern Australia (*57*, *59*). In addition, variables such as submarine spring sapping, which can induce slope failure (*40*, *60*), and sediment delivery by shelf currents are not globally quantified but can be incorporated as data become available. However, a general proxy for submarine spring sapping is implicitly accounted for in the submarine groundwater discharge model, which is included as a predictor (table S1) (*30*).

Last, several predictor variables fluctuate over time as climatic and tectonic variations drive shifts in water and sediment discharge over geological timescales. While these changes are not yet globally quantified, our approach relies on present-day data and assumes that their relative values have remained stable over time—an assumption that may not always hold. For example, sediment discharge (Qs) has varied across the past glacial cycles (*61*). In particular, the Gulf of Lion received varying amounts of sediment from the Alps through the Rhône and adjacent river systems during the past glaciations. Here, the shelf edge’s depth at ~120 m is higher compared to other continental margins (*62*) and at a similar elevation as sea levels during glacial times. This allowed rivers to extend to the shelf edge, contributing to submarine canyon development following sea level rise (*63*). Similarly, along southeastern Australia, increased carbonate sedimentation rates during the Pliocene-Pleistocene transition led to enhanced relief and slope instability, potentially fostering canyon formation (*15*, *64*). In addition, offshore Mauritania, canyon incision has been attributed to a combination of shelf incision by a Pliocene-Pleistocene river system and sand contribution from eolian processes (*65*), not accounted for in existing sediment transport models (*66*). However, most canyons in this study remained disconnected from river systems even during extreme low–sea level conditions, suggesting that slope-driven processes play a dominant role in their formation (fig. S8). Moreover, the temporal dynamics of canyon evolution present additional challenges for our model, which may overlook the complex interplay between erosive and depositional processes that occur over millennial to million-year timescales (*63*). Submarine canyons do not form instantaneously but evolve through multiple stages of incision, sediment infill, and reactivation, driven by variations in sea level, tectonic activity, and sediment supply. As a result, determining the precise timing of initial canyon formation remains inherently difficult, and canyon formation age cannot be incorporated into our model. In summary, many key variables influencing canyon evolution—such as structural controls, erodibility of marine substrate, and long-term variations in sediment flux—are either unavailable at the global scale or lack the resolution necessary for robust predictive modeling. While our approach provides valuable insights into global-scale controls on canyon distribution, future advancements could be achieved by incorporating these potentially critical variables and their temporal dynamics as these data become available, for example, through global numerical simulations (*67*).

In conclusion, processes of submarine canyon formation are inherently complex and make it difficult to study the phenomenon using physical models. Our statistical approach addresses this complexity by identifying key correlations between canyon occurrence and both terrestrial and marine variables while acknowledging that these correlations do not imply direct physical causation. Despite its simplicity, our model captures global canyon distribution remarkably well—particularly for slope-confined canyons—using only one to two key predictor variables. Hence, it provides a valuable framework for establishing a global “baseline” canyon density, guiding future research toward a more mechanistic understanding of canyon development and enhancing predictions of canyon formation throughout Earth’s history.

Our results demonstrate that high submarine canyon occurrences are strongly associated with steeper continental slopes, suggesting that their initiation is primarily driven by mass wasting processes along the slope, followed by erosion from sediment density currents triggered by these failures. However, the hypothesized link between the formation of slope-confined canyons and terrestrial geomorphic processes is not supported by our analysis at the global scale. Instead, submarine canyon distribution appears to be predominantly controlled by tectonic deformation, basin-floor subsidence, margin uplift, and the shear strength of margin-forming material. Canyons start interacting with shelf processes as they erode into the continental shelf. Our findings further indicate that once a canyon incises into the shelf, it limits the ability of nearby canyons to do the same, due to its preferential position for capturing sediment flows. Terrestrial processes become relevant once canyon heads approach the shoreline, with sediment durability and water discharge likely playing key roles in maintaining shore-canyon connectivity throughout glacial-interglacial sea level fluctuations (*12*).

With the advent of global landscape evolution models capable of reconstructing past landscapes and seascapes (*67*), our findings provide critical insights into submarine canyon distribution and offer a foundation for estimating canyon occurrence, continent-ocean connectivity, and the transport and burial of sediment and associated organic carbon throughout Earth’s history (*7*, *68*). These estimations represent essential puzzle pieces in understanding and quantifying the role of terrestrial organic carbon burial by marine sedimentary systems as a driver of long-term global climate regulation.

## MATERIALS AND METHODS

### Submarine canyon occurrence

Large-scale submarine canyons (water depth range, ≥1000 m; incision, ≥100 m) were previously mapped (*3*, *9*) on a global scale using the SRTM30_PLUS data with a 30–arc sec resolution (*4*, *10*, *27*) and the Australian Bathymetry and Topography Grid (*69*). At the global scale, the spatial occurrence of canyons can be conceptualized as a spatial point pattern. We therefore used the canyon data provided (*4*) and calculated the centroid of each canyon polygon. Moreover, we used the mapped continental slope from the same publication (*9*) and calculated the centerline. We clipped all canyon centerpoints within the continental slope polygon used in this study and projected the centerpoints onto the continental slope centerline (fig. S1) with a snapping distance of 100 km. We treated these as point patterns along a linear network (*25*, *26*), because—as submarine canyons traverse the continental slope—only one canyon can occur along a lateral stretch of the continental slope. We restricted our analyses to the main continents between 50°N and 50°S and excluded islands. In addition, we excluded margins that are highly influenced by salt tectonics and/or glaciation (e.g., Gulf of Mexico, the US East Coast until 40°S, and the Chilean fjord region).

### Quality of the bathymetric data and its impact on canyon maps

The SRTM30_PLUS Ocean bathymetry was derived from a satellite gravity model, and the gravity-to-topography ratio was calibrated using 298 million edited soundings from various sources (*27*). The quality of the bathymetric data strongly depends on the density of soundings in a certain area. In addition, canyons around Australia have been mapped on the Australian Bathymetry and Topography Grid (*69*), which was down-sampled to the same grid size as the SRTM30_PLUS grid (*4*). However, the Australian grid is based on a large number of surveys, especially around the shelf and the continental slope of Australia (*69*). Hence, we expect that data quality affects the number of canyons mapped in a certain area. To account for the dependence of canyon maps on data quality, we calculated a grid that contains the average number of ship tracks [from which the soundings were derived (*4*)] per 10-km grid cell using the aggregate function of TopoToolbox (*70*). To further account for the high data quality of the Australian grid in the areas, where continuous surveys were available, we inserted the maximum ship-track density of 1 into the ship-track density grid. We used the ship-track density grid as an additional predictor variable in the point pattern models (see fig. S2 for the dependence of canyon density on ship-track density).

### Explanatory variables

We computed eight marine variables, seven terrestrial variables, and one categorical spatial explanatory variable (also “covariates” or predictors) from globally available data, additional to the ship-track density explained earlier (Fig. 1). All explanatory variables, their computation, and data sources are listed in table S1. Values of the terrestrial variables were originally calculated from global datasets (*27*, *32*–*36*) and obtained from previous publications in which values were calculated for each river outlet using a weighting scheme that included the inverse distance and river catchment area (*12*, *49*). To project terrestrial variables to the continental slope, we first calculated all distances *d*_i_ between the river outlets and all pixels along the continent’s coastlines.

These distances *d*_i_ together with the catchment areas *A*_i_ of each river outlet served as weights in the same distance-weighted averaging approach used previously (*12*, *49*). Specifically, we calculated the weights ω_i_ as

ωi =Aidi3∑iAidi3 (1)

Terrestrial explanatory variables were then projected or mapped onto the continental slope using distance transform (bwdist function in MATLAB), assigning the values of the closest pixel of each dataset to each pixel on the continental slope.

Marine explanatory variables were extracted from global datasets (*4*, *27*, *31*, *71*, *72*) and were not weighted. The details of which marine variable is based on which dataset are listed in table S1. The continental shelf (slope) inclination was computed using the outline of shelf (slope) shapefile (*4*). Laplace interpolation was applied between shelf (slope) boundaries to create a smoothed shelf (continental slope) without submarine canyons. For the shelf, these boundaries are the coastline and the shelf edge, between which interpolation was performed. For the continental slope, the boundaries are the shelf edge and the toe of the slope. The idea behind this approach is to minimize the discrete Laplacian of the interpolated function, ensuring a smooth transition between known and fixed data points (*73*). We computed the mean inclination of the smoothed shelf and slope using the arcslope function in TopoToolbox (*70*). Sediment thickness along the continental margin was corrected for submarine canyons by cutting the thickness dataset (*30*) with the shapefiles of the mapped submarine canyons and linearly extrapolating over the missing values to avoid inherent correlations where low thicknesses correlate with high canyon incision. When explanatory variables were constrained to the positive real space (*R*+) or the simplex (*S*), the explanatory variables were transformed to the real space (*R*) (*74*, *75*). All numerical variables were scaled.

### Point pattern analysis

To explore the global distribution of submarine canyons, we conceptualize their locations as points along the centerlines of the continental slopes. This is achieved by identifying the centroid of each canyon polygon and projecting these centroids onto the centerline of the continental slope (fig. S1). This approach simplifies the analysis by treating the canyon locations as points distributed along a linear network of continental slope centerlines along the world’s major continents, which spans 104,200 km. While this method omits detailed information about individual canyon features (e.g., size, shape, or exact location), it enables a broader understanding of canyon distribution and density on a global scale, providing insights into the factors controlling canyon formation.

We tested whether slope-confined and shelf-incised canyon densities follow homogeneous Poisson point processes (i.e., are completely randomly distributed along the linear network). Kolmogorov-Smirnov tests of complete spatial randomness (cdftest function of TopoToolbox with the covariate ship-track density) rejected the null hypothesis that submarine canyons of any type are distributed randomly along the major continental margins with *P* values of 9.4 × 10^−56^, 3.3 × 10^−50^, and 1.9 × 10^−13^ for all slope-confined and shelf-incised submarine canyons, respectively (*P* << 0.05 indicates that the null hypothesis of complete spatial randomness is rejected). Hence, all canyon types are assumed to follow an inhomogeneous point process, even if their dependence on data quality (covariate “ship-track density”) is considered.

We modeled submarine canyon occurrence along the continental slope as a spatial point pattern on a linear network (*25*, *26*). A spatial point pattern is a dataset of the observed spatial locations of occurrences (fig. S1). The goal of point pattern analysis is to infer the mechanisms (the spatial point process) that generate the spatially variable densities of the points. To attain this goal, we use both exploratory, nonparametric (kernel density estimators and dependence estimators) and parametric (hypothesis testing and inhomogeneous Poisson process models) modeling techniques. To obtain average density of submarine canyon densities along the continental slope (Fig. 3, A and C), we applied a kernel estimation to each unit length of the linear network with a Gaussian kernel and a bandwidth of 200 km.

We used the numeric class “Point Pattern on Stream networks” of the MATLAB-based terrain analysis software TopoToolbox (*26*), which is strongly influenced by the R package “spatstat” (*24*) and uses functions from the BayesReg toolbox (*76*, *77*) to derive a parametric model of submarine canyon densities (λ) using inhomogeneous Poisson process models. This class of models is defined by its intensity function (*u*), with *u* being the network-attached coordinates along the continental slope centerline (which we treated as a stream network). The most frequently used model is the log-linear model

$$\lambda \left(u\right)=e^{\beta_{0}+\beta X\left(u\right)}$$ (2)

where **X** is the matrix of 16 explanatory variables and ship-track density (17 variables in total) (table S1), β_0_ is the intercept, and β is a vector of 17 regression coefficients describing the relationship between each explanatory variable and the response variable (so-called first-order effects). Before regression modeling, all explanatory variables that are compositional (i.e., carrying relative rather than absolute information) or confined to the positive real space were log-transformed to the real space (*74*, *78*, *79*). All variables were scaled to have zero mean and an SD of one (table S1).

To minimize the number of explanatory variables and to avoid overfitting, we used a stepwise model selection based on the AIC. This approach iteratively omits explanatory variables from the model if their inclusion in the model does not enhance model performance expressed in a lowering of the AIC by 10. The function ploteffects (in TopoToolbox, effectfun in spatstat) visualizes the individual effects of a log-linear point process model (Fig. 4, B and C).

To assess the robustness of our results across approaches, we used a Bayesian approach to analyze log-linear models. Bayesian statistics apply probabilities to statistical problems, offering a way to learn from new data to update prior beliefs while accounting for uncertainties (*80*). A frequentist approach to penalization is Lasso regression, which uses a penalty term to shrink small regression coefficients to zero (hence reducing or eliminating unimportant explanatory variables) (*81*). In Bayesian penalized regression, penalization is incorporated through the choice of prior, and we used a Lasso prior to avoid overfitting using the function bayesloglinear in TopoToolbox, which is based on the BayesReg toolbox (*76*, *77*, *82*). Last, we quantified the importance of each explanatory variable adopting the Bayesian feature–ranking algorithm (*77*). The rank corresponds to the strength of the association between the explanatory variable and the response and is based on the 75th percentile of the complete set of rankings for each posterior sample (tables S2 and S3).

### Operational join-count statistics to detect remaining canyon clustering (residual analyses)

We used operational join-count statistics (*39*) to detect remaining canyon clustering, an alternative form to analyzing residuals. Using this approach, we identified remaining clusters or “hotspots” of slope-confined and shelf-incised canyon occurrence that are not explained by the first-order controls considered in the Bayesian model. Canyon occurrence (of either canyon type) is a binary variable (canyon occurrence, *x*_i_ = 1; no canyon, *x*_i_ = 0). In the univariate case (only one canyon type is considered), the so-called BB (black-black) join count assesses whether a canyon of a certain type (i.e., *x*_i_ = 1) is surrounded by more locations with canyons than would be the case under the null hypothesis. In our case, the null hypothesis refers to our predicted densities obtained from the Bayesian model. Join counts thus detect the remaining canyon clusters, whose densities exceed those predicted by our inhomogeneous Poisson point process model.

A BB join count takes the form of

$$\text{BB}=\sum_{i}\sum_{j}w_{\mathrm{ij}}x_{i}x_{j}$$ (3)

with *w_ij_* being a matrix of weighted distances (*d*) between canyons at locations *i* and *j* (with 1/*d_ij_*). Low distances indicate that canyon locations adjoin. We calculated the BB join-count statistics for slope-confined canyons, shelf-incised canyons, and shelf-incised canyons surrounded by slope-confined canyons.

We calculated the probability (*p*) that a canyon is either slope confined or shelf incised based on their predicted densities by the Bayesian model (λ_slope-confined_ and λ_shelf-incised_; fig. S4).

$$p_{\text{slope}-\text{confined}}=\frac{\lambda_{\text{slope}-\text{confined}}}{\lambda_{\text{slope}-\text{confined}}+\lambda_{\text{shelf}-\text{incised}}}$$ (4)

$$p_{\text{shelf}-\text{incised}}=\frac{\lambda_{\text{shelf}-\text{incised}}}{\lambda_{\text{shelf}-\text{incised}}+\lambda_{\text{slope}-\text{confined}}}$$ (5)

We simulated the 1000 BB join counts under the assumption that the canyons are distributed according to the inhomogeneous Poisson point process model (null hypothesis) and calculated the 95 percentiles of these simulations (*BB95*_sim_). We compared the actual BB join count to the simulated 95 percentiles and visualized the difference between the two as

$${\text{BB}}_{\text{canyon}}-\text{BB}95{\text{sim}}_{\text{canyon}}$$ (6)

[see Fig. 5 (A and B)].

Last, we tested the hypothesis that shelf-incised canyons are surrounded by more slope-confined canyons than predicted by the inhomogeneous Poisson point process model. We calculate the BB join count (Eq. 3) with the weighted *w_ij_* between shelf-incised canyons at locations *i* and slope-confined canyon neighbors at location *j*. We analogously simulated 1000 BB join counts under the assumption that the slope-confined canyons are distributed according to the Bayesian model and compared the simulated 95 percentiles to the actual BB join count (Fig. 5C). All data and MATLAB codes are available as a data publication (*83*).

## Supplementary Materials

### This PDF file includes:

Figs. S1 to S8 Tables S1 to S3 References
