# Supplementary material for: Seafloor slopes control submarine canyon distribution: A global analysis
Source: Sci Adv. 2025 Jun 11;11(24):eadv3942. doi: 10.1126/sciadv.adv3942 (PMC13109954; doi:10.1126/sciadv.adv3942)
Supplement: Supplementary file 1 — Figs. S1 to S8 Tables S1 to S3 References [file sciadv.adv3942_sm.pdf]

Science Advances
AAAS

Supplementary Materials for

Seafloor slopes control submarine canyon distribution: A global analysis

Anne Bernhardt and Wolfgang Schwanghart

Corresponding author: Anne Bernhardt, anne.bernhardt@fu-berlin.de

Sci. Adv. **11**, eadv3942 (2025)
DOI: 10.1126/sciadv.adv3942

This PDF file includes:

Figs. S1 to S8
Tables S1 to S3
References

Supplementary Figures and Tables

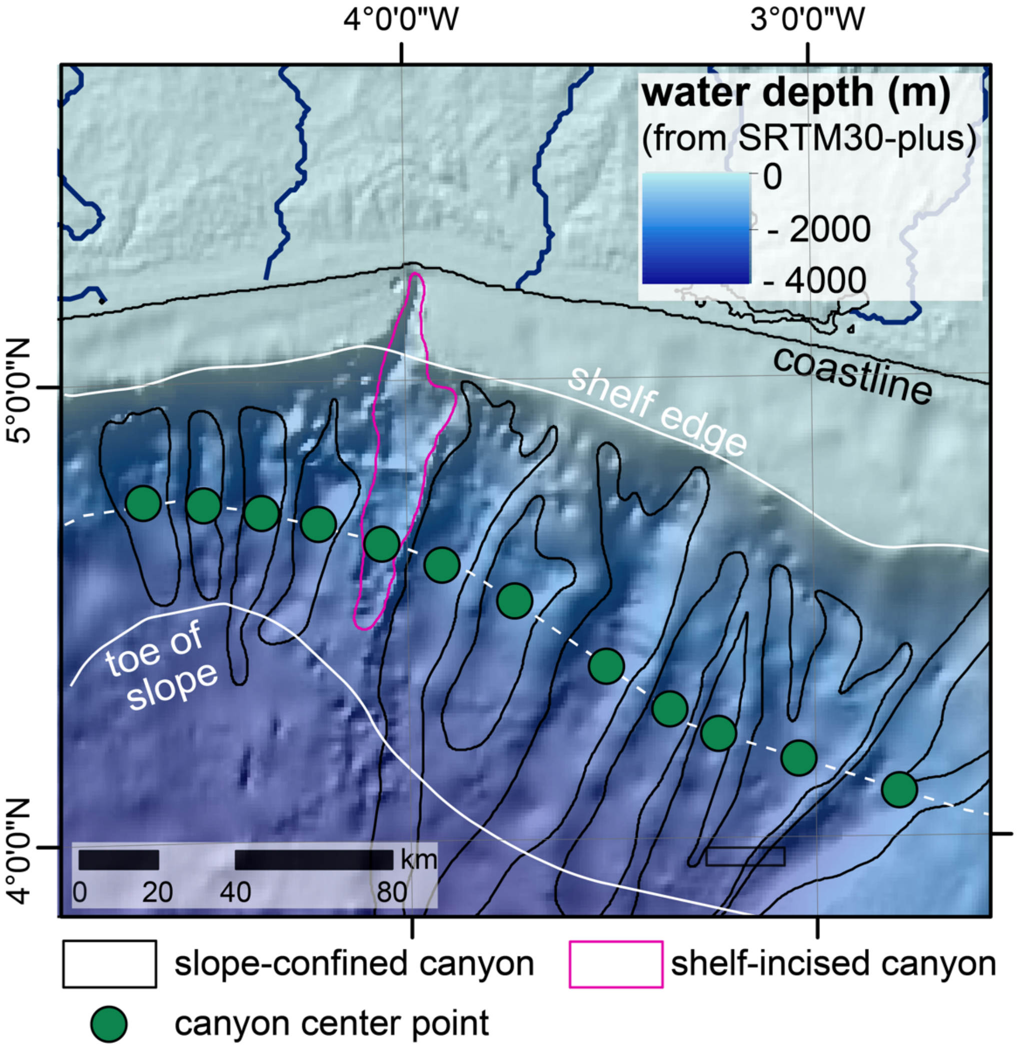

Fig. S1. Distribution of large slope-confined and shelf-incised canyons (4) offshore the Ivory Coast. Most canyons are classified as slope-confined and one canyon incises into the shelf. The white solid lines show the outline of the continental slope, the stippled white line represents the centerline of the continental slope. Canyon center points were computed and then collapsed onto the centerline of the continental slope. As only one canyon can occur along each stretch of the continental slope, the canyon center point were treated as point patterns along a linear network(*26*, *27*) (= the continental slope centerline).

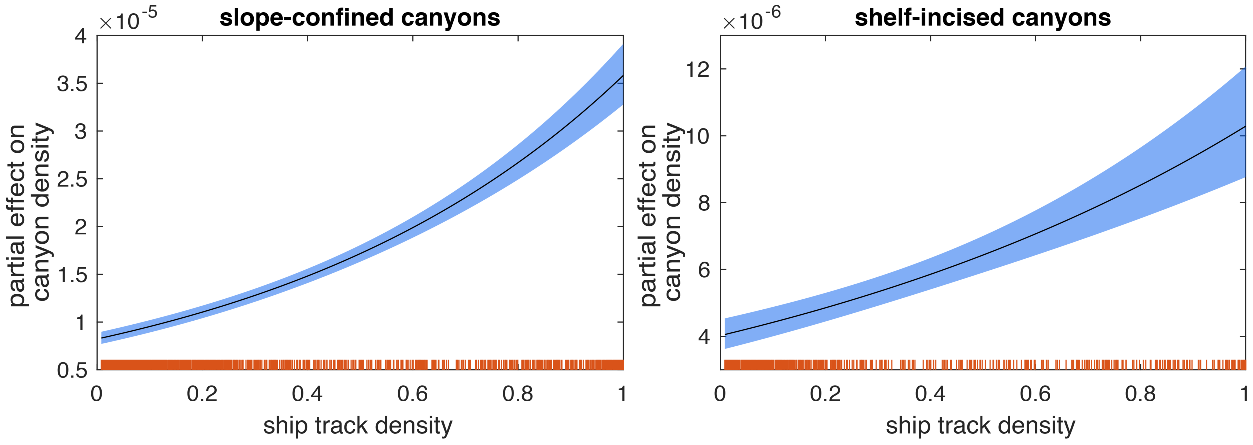

Fig. S2. The partial effect of ship-track density on slope-confined canyon density (left panel) and on shelf-incised canyon density (right panel). These plots show how canyon density varies as a function of the ship-track density (in ship tracks per grid cell, which have provided bathymetric soundings to the global bathymetric map). Hence, this predictor provides a measure for bathymetric data quality. Canyon density increases with an increase in ship-track density showing that mapped canyon density strongly depends on the quality of the bathymetric data used for canyon mapping. Orange lines at the bottom of the plot indicate canyon (point) locations. To account for the variable data quality in the model, ship-track density is included into the model as an explanatory variable.

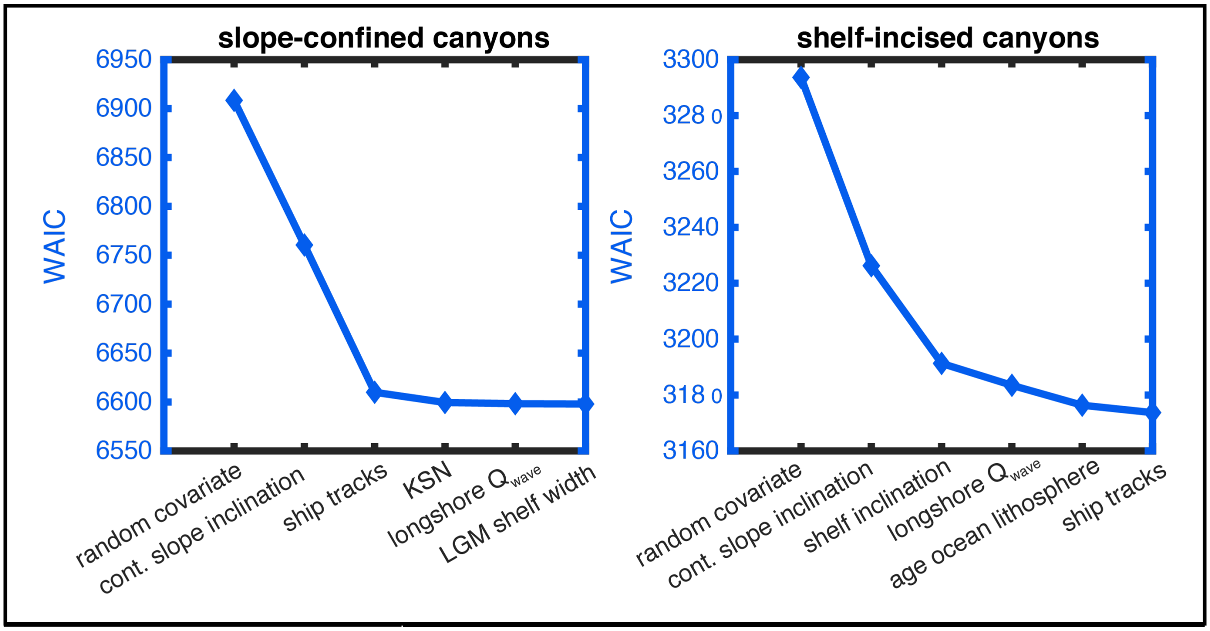

Fig. S3. Development of the Widely Applicable Information Criterion or Watanabe-Akaike information criterion (WAIC) of the inhomogeneous Poisson Point Process Model when adding additional explanatory variables for slope-confined and shelf-incised canyons. In simple terms, the WAIC is a method for comparing different models by evaluating how well they fit the data while accounting for model complexity. It balances goodness-of-fit and model complexity (i.e., the number of predictor variables used), helping to identify the best-fitting model while avoiding overfitting. Moreover, the WAIC (*82*) extends AIC within a Bayesian framework, leveraging the full posterior distribution for improved performance in hierarchical and complex models. Note that the variable ship-track density refers to the dependence of canyon densities on bathymetric data quality, but not to a physical process. Incorporating continental slope inclination into the model yields the highest reduction in WAIC for both canyon types (WAIC: -148 for slope-confined canyons, WAIC: -67 for shelf-incised canyons).

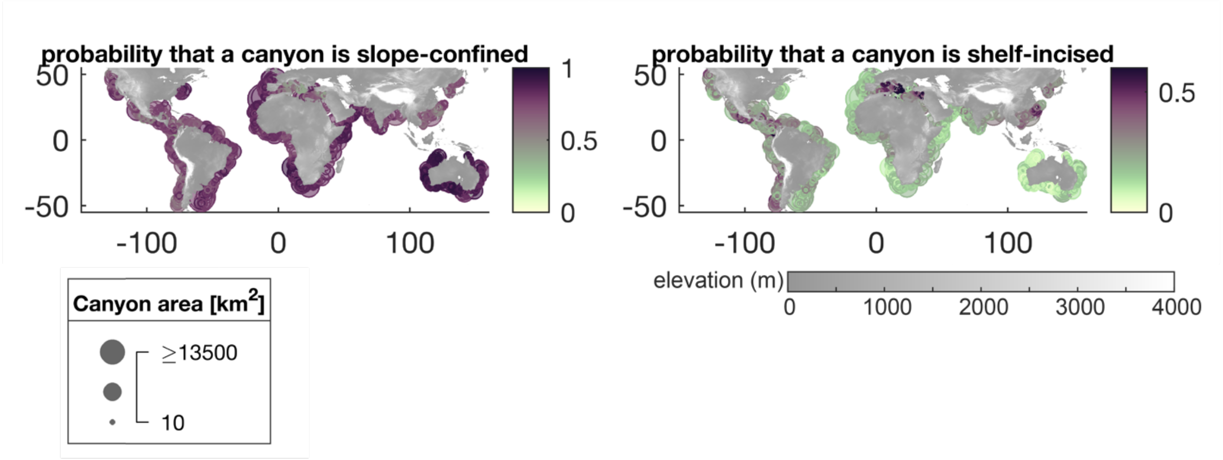

Fig. S4. Probability that a canyon at a specific location is slope-confined or shelf-incised. For details on the computation, see Equations 4 and 5 in Methods.

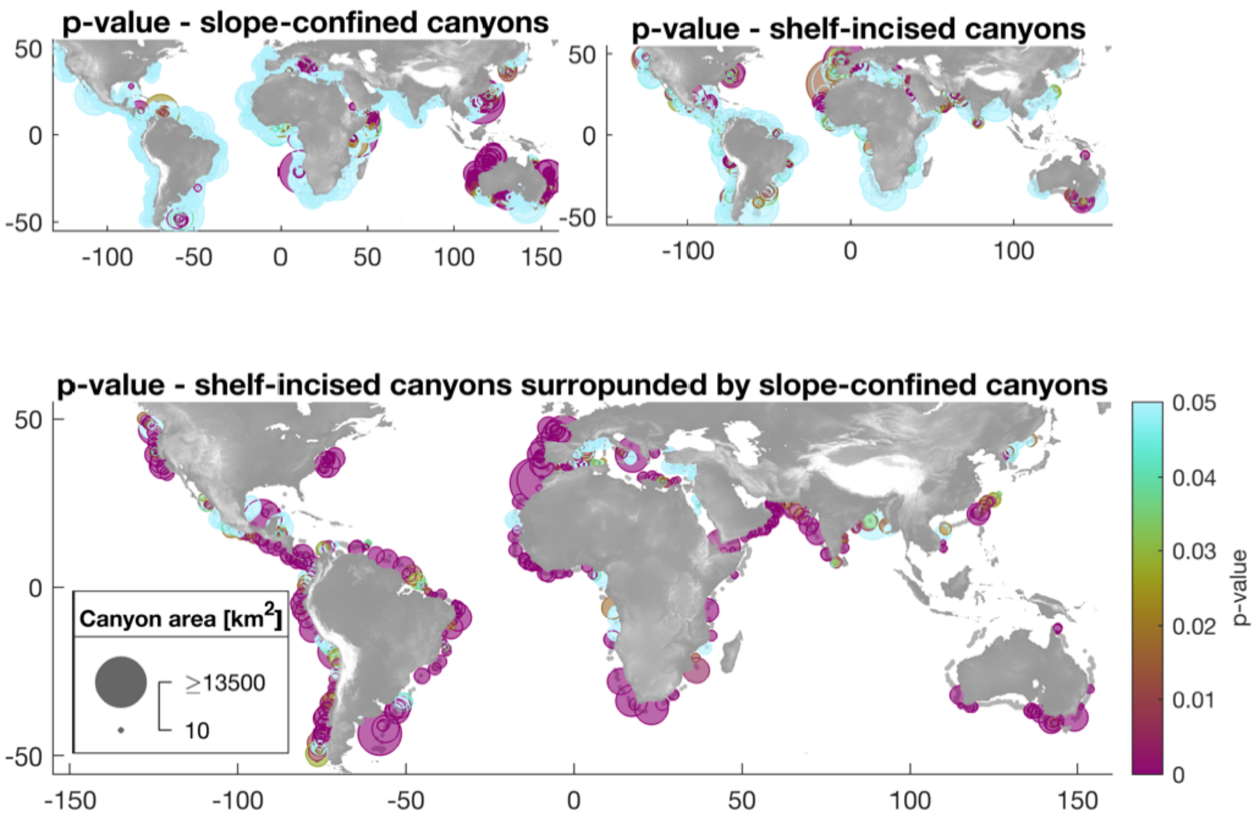

Fig. S5. P-values associated with the remaining canyon hotspots or clusters shown in Figure 5 in the main manuscript. These show the probability that we erroneously accept the alternative hypotheses that the slope-confined and shelf-incised canyons occur more clustered or that the shelf-incised canyons are surrounded by more slope-confined canyons than predicted by the Bayesian penalized regression. For elevation color scale (greyscale) compare to Fig. 3 and 5 in the main text.

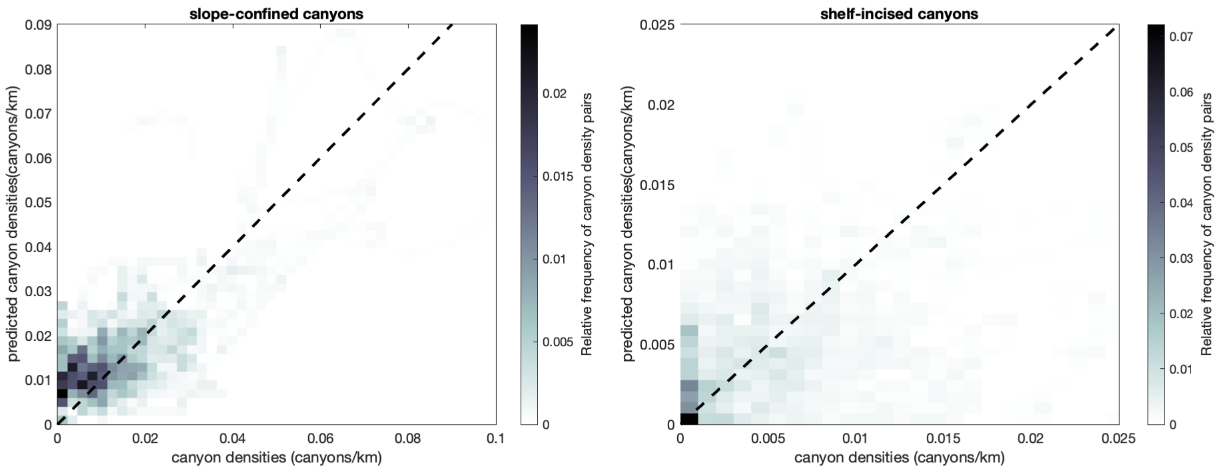

Fig. S6: Joint distribution of observed and predicted canyon densities. The 2D histogram visualizes the relative frequency of canyon density pairs, with observed values on the x-axis and model-predicted values on the y-axis. Bin counts are normalized so that their total sums to one, and the color scale represents the relative frequency per bin. The dashed black 1:1 line indicates perfect agreement between observations and model predictions.

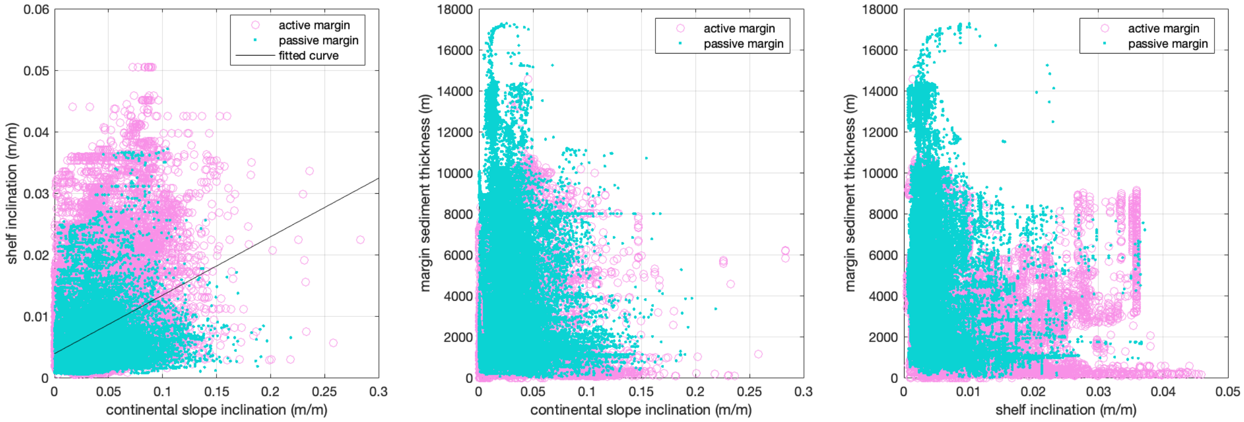

Fig. S7. Scatter plots of various predictor variables. Left panel shows the cross-correlation of shelf and continental slope inclination. The best-fit line follows a linear relationship ($y = 1.78x + 0.02$) with an R^2 value of 0.17, indicating a very weak dependence between shelf and continental slope inclination. Active margins exhibit the highest continental slope inclinations and significantly steeper shelf inclinations compared to passive margins. Middle and right panels show scatter plots of continental slope inclination and shelf inclination against margin sediment thickness, respectively. Both inclinations show no correlation with margin sediment thickness (both R^2 values < 0.05).

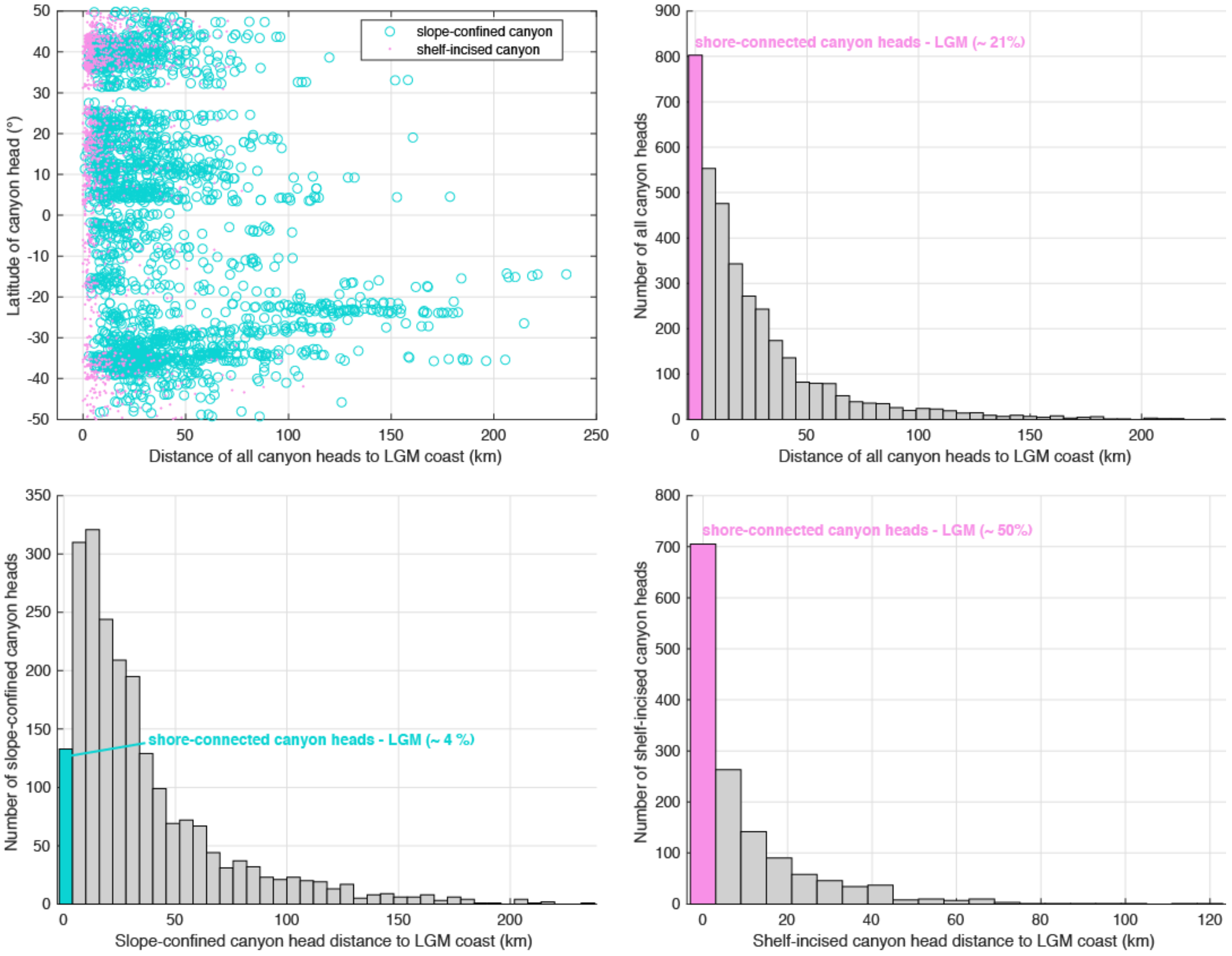

Fig. S8: Canyon Head Proximity to the Last Glacial Maximum (LGM) shoreline. These data are derived from previous research(*50*) and have been filtered for the canyons analyzed in this study. Note that in this approach, the heads of submarine canyons were mapped and complex canyon systems can contain multiple canyon heads. Left Panel (first row): The distance of each canyon head from the simulated LGM shoreline at 120 m below present sea level is plotted against latitude, illustrating the spatial distribution of canyon heads relative to the paleo-coastline. Right Panel (first row): Frequency of canyon heads in 6 km distance bins from the LGM shoreline, demonstrating that a substantial proportion (~80%) of canyons were not connected to the shoreline during the extreme sea-level lowstand related to the LGM. Left Panel (second row): Frequency of slope-confined canyon heads in 6 km distance bins from the LGM shoreline, demonstrating that a substantial proportion (~96%) of slope-confined canyons were not connected to the shoreline during the extreme sea-level lowstand during the LGM. Right Panel (second row): Frequency of shelf-incised canyon heads in 6 km distance bins from the LGM shoreline, demonstrating that a substantial proportion (~50%) of shelf-incised canyons were connected to the shoreline during the extreme sea-level lowstand related to the LGM.

Explanatory variable	Unit	Description & computation	Transformation to real space & scaling	Data source	Reason for inclusion
**variable accounting for data quality**					
ship-track density (shiptrack)	mean ship tracks per cell	average number of ship tracks (from which the echo soundings were derived) per 10 km grid cell using the aggregate function of TopoToolbox; around Australia, where continuous surveys were available, maximum ship track density of 1 was used	transformation from R+ with upper and lower bounds (max & min): x=(shiptrack-min)/(max-min) shiptrack_transformed = log(x/1-x) min=0, max=1 scaled	Becker et al. (28) Whiteway et al. (70)	We included ship track density as a predictor to account for bathymetric data quality.
**categorical variables**					
continental margin type (passive)	unitless	passive and tectonically active continental margins	no transformation	Harris et al. (4)	Different canyon densities between the two margin types have been proposed.
**terrestrial variables**	All values from Bernhardt and Schwanghart (12, 50) were projected onto the coastline using the weighting factor $\lambda_i = \frac{A_i}{d_i^3}/(\Sigma_i \frac{A_i}{d_i^3})$. Projection onto the continental slope using a nearest neighbor approach.				
mean gradient of rivers in onshore catchments (present day) (rivgrads)	unitless	weighted mean gradients from each river outlet were taken from Bernhardt and Schwanghart (12, 50) gradient and meanupstream function of TopoToolbox	log-transformed scaled		Characterization of river catchments adjacent to canyons.
mean river steepness index in onshore catchment (present day) (KSN)	unitless	ksn and meanupstream function of TopoToolbox	log-transformed scaled		Characterization of river profiles adjacent to the canyon.
mean annual discharge of onshore catchment (Qw)	m^3/s	flowacc function of TopoToolbox	log-transformed scaled	Fekete et al. (33)	High Qw may foster high sediment supply to the canyon head.
modeled suspended sediment flux (pre-human) (Qs)	kg/s	Qs at river outlets was assigned to the HydroSHEDS stream network using the knnsearchlatlon & matchpairs function of TopoToolbox/ MATLAB	log-transformed scaled	Syvitski and Kettner (34) (BQART model)	High Qs may foster canyon-head erosion.
peak ground acceleration (GSHAP)	m/s^2	peak ground acceleration from the Global Seismic Hazard Map of the Global Seismic Hazard Assessment Program (GSHAP), which depicts PGA with a 10% probability of exceedance in 50 years- was assigned to each river outlet using the flowacc function of TopoToolbox	log-transformed scaled	Shedlock et al. (35)	As a proxy for tectonic activity, intense seismic shaking may trigger erosive turbidity currents and mass failure.
weighted global erodibility index (ero)	unitless	GEroID ranges from 1.0 (low erodibility) to 3.2 (high erodibility), area-weighted mean of GEroID for each drainage basin was assigned to each river outlet using the upslopestats function of TopoToolbox	transformation from R+ with upper and lower bounds (max & min): x=(ero_index-min)/(max-min) ero_trans = log(x/1-x) min=0, max=3.2 scaled	Moosdorf et al. (36)	Sediment supply from low-erodibility bedrock may enhance erosion of the canyon head and floor.
The ratio of the 90th to 50th percentile of the mean annual rainfall. (TRMM90)	unitless	ratio measures the extremity of rainfall; high values indicate extreme rainfall events; area-weighted mean of the 90th-to-50th percentile ratio of the mean annual rainfall for each catchment was assigned to each river outlet using the upslopestats function of TopoToolbox.	log-transformed scaled	Boers et al. (37)	Extreme rainfall events may lead to high instantaneous sediment supply and potentially trigger hyperpycnal flows that enter canyon heads.

marine variables	marine explanatory variables are not weighted				
gradient of the adjacent shelf (shelf_gradient)	unitless (m/m)	computed outline of continental shelf (Harris et al. (4)), Laplace interpolation between shelf boundaries to create a smoothed shelf without submarine canyons, computed mean gradient of the smoothed shelf using the arcslope function of TopoToolbox	log-transformed	SRTM30_PLUS Becker et al. (28), Harris et al. (4)	High shelf gradient can promote erosive turbidity currents that foster canyon-head incision.
gradient of the adjacent continental slope (cs)	unitless (m/m)	analogous to shelf gradient but using the continental slope shapefile of Harris et al. (4)	log-transformed	SRTM30_PLUS Becker et al. (28), Harris et al. (4)	High slope gradient can promote slope failure and erosive turbidity currents.
mean shelf width (present day) (shelf_width)	km	calculated the shelf width using the mapped shelf of Harris et al. (4) (using *bwdist* in MATLAB)	log-transformed	Harris et al. (4)	Narrow shelves may facilitate canyon incision into the shelf.
mean shelf width (LGM) (LGMshelf)	km	identical calculation to the present-day shelf width, but based on SRTM30_PLUS DEM, where 120m were added to the elevation data.	log-transformed	SRTM30_PLUS Becker et al. (28), Harris et al. (4)	Narrow shelves may facilitate canyon-to-shore connection.
modeled fresh submarine groundwater discharge (SGD)	m^2/yr	modeled fresh submarine groundwater discharge (SGD) extrapolated to the continental slope using *bwdist*	log-transformed scaled	Luijendijk et al. (30)	Submarine groundwater seepage may lead to seafloor failure and canyon initiation.
sediment thickness along continental margin (sedthick)	km	GlobSed: global 5-arc-minute total sediment thickness grid for the world's oceans and marginal seas. Canyons were cut from the thickness maps and values were interpolated into the voids by a nearest neighbour approach	log-transformed scaled	Straume et al. (31)	Proxy for sediment flux over geologic time scales.
age of the continental lithosphere (agelitho)	million years	ages of the oceanic lithosphere were extrapolated to the continental slope using *bwdist*	log-transformed scaled	Seton et al. (32)	Submarine canyon development takes time; thus, adjacent oceanic lithosphere age can serve as a proxy for passive margin age.
longshore wave-induced sand transport (LST or Qwave)	m^2/yr	estimate of the potential maximum annual flux of sediment transport along the coast driven by waves	log-transformed scaled	Regard et al. (67)	Submarine canyons may be fed by longshore transport, promoting shelf incision at canyon heads.

Table S1. Predictive variables. List, data sources(4, 12, 28–30, 32–37, 50, 72, 73) and explanation of the 17 predictive variables (16 explanatory variables and including ship-track density as a data quality proxy).

```
===================================================================================================
|                        Bayesian Penalised Regression Estimation ver. 1.91                      |
|                            (c) Enes Makalic, Daniel F Schmidt. 2017-20                          |
===================================================================================================
Bayesian logistic lasso regression                                      Number of obs   =    51708
                                                                        Number of vars  =       17
MCMC Samples   =   1000                                                 Log. Likelihood =  -6579.4
MCMC Burnin    =   1000                                                 Pseudo R2       =   0.0475
MCMC Thinning  =      5                                                 WAIC            =   6593.5

---------------+-----------------------------------------------------------------------------------
     Parameter |   mean(Coef)    std(Coef)        [95% Cred. Interval]       tStat    Rank       ESS
---------------+-----------------------------------------------------------------------------------
            cs |    10.96123      0.84953       9.21719     12.63376      12.895       1 **    57.1
     shiptrack |     0.08319      0.00518       0.07367      0.09331      16.047       1 **    74.4
           KSN |    -0.08848      0.03602      -0.15813     -0.01889      -2.462       3 **    53.0
           LST |    -0.27562      0.17373      -0.62224      0.03537      -1.572       4 *     38.0
      LGMshelf |     0.04246      0.02696      -0.00457      0.09902       1.638       5 *     48.6
            Qs |    -0.01867      0.01743      -0.05508      0.01228      -1.111       6 *     65.3
      agelitho |     0.03586      0.03157      -0.01712      0.10230       1.190       6 *     46.1
               |
       passive |
            2  |     0.09646      0.08148      -0.03799      0.26714       1.236       6 *     55.6
            Qw |    -0.01244      0.01593      -0.04882      0.01448      -0.848       9 *     75.7
   shelf_width |    -0.02429      0.03683      -0.11003      0.03696      -0.776      10 *     70.0
               |
      rivgrads |    -0.49384      0.63518      -1.92786      0.65043      -0.831      11 *     57.0
         GSHAP |     0.00223      0.06008      -0.11593      0.12018       0.027      12        70.3
           SGD |     0.00238      0.00920      -0.01658      0.01989       0.230      12        44.3
        TRMM90 |     4.98814     30.63514     -52.77768     70.00842       0.223      12        61.7
           ero |     0.01880      0.02657      -0.03282      0.07615       0.742      12 *      43.7
      sedthick |    -0.01313      0.02828      -0.07462      0.03793      -0.555      12        66.4
shelf_gradient |    -1.37284      4.90163     -12.27602      6.88108      -0.355      12        72.2
         _cons |    -3.48809      0.35762      -4.20101     -2.79439           .       .           .
---------------+-----------------------------------------------------------------------------------
```

Table S2. Results of Bayesian loglinear model approach for slope-confined canyons. Abbreviations for explanatory variables are listed in table S1.

Bayesian Penalised Regression Estimation ver. 1.91
(c) Enes Makalic, Daniel F Schmidt. 2017-20

Bayesian logistic lasso regression				Number of obs	=	51708
				Number of vars	=	17
MCMC Samples	=	1000		Log. Likelihood	=	-3138.2
MCMC Burnin	=	1000		Pseudo R2	=	0.0469
MCMC Thinning	=	5		WAIC	=	3153.6

Parameter	mean(Coef)	std(Coef)	[95% Cred.	Interval]	tStat	Rank		ESS
cs	10.68630	1.36508	8.11383	13.43084	7.854	1	**	21.5
shelf_gradient	41.42339	7.98439	26.13870	57.18085	5.185	1	**	38.3
LST	-0.82227	0.26070	-1.34055	-0.30064	-3.144	3	**	31.5
shiptrack	0.03907	0.01146	0.01477	0.06108	3.398	3	**	30.1
SGD	0.05063	0.01785	0.01549	0.08353	2.831	5	**	30.8
agelitho	-0.13616	0.04951	-0.23285	-0.03419	-2.764	5	**	28.0
shelf_width	0.12950	0.06487	0.01474	0.26064	2.025	5	**	23.3
Qw	0.04914	0.02865	-0.00422	0.10384	1.744	8	*	31.1
LGMshelf	-0.06321	0.04449	-0.15468	0.01322	-1.441	9	*	22.9
KSN	-0.04302	0.05131	-0.16116	0.04238	-0.928	10	*	34.2
Qs	0.01354	0.02664	-0.03250	0.06870	0.559	10		38.9
ero	-0.05688	0.03482	-0.11909	0.01573	-1.578	10	*	39.8
passive								
2	-0.09831	0.11556	-0.34102	0.10260	-0.935	10	*	30.6
rivgrads	0.81408	0.90593	-0.72466	2.74958	1.006	10	*	38.9
sedthick	0.05146	0.04418	-0.02524	0.14087	1.185	10	*	26.7
GSHAP	-0.04749	0.08805	-0.22998	0.11277	-0.585	16		29.1
TRMM90	-39.50770	50.45857	-145.54811	48.02950	-0.841	16	*	30.0
_cons	-5.44356	0.60074	-6.65661	-4.34291	.	.		.

Table S3. Results of Bayesian loglinear model approach for shelf-incised canyons. Abbreviations for explanatory variables are listed in table S1.

REFERENCES AND NOTES

1. M. Pierdomenico, A. Bernhardt, J. T. Eggenhuisen, M. A. Clare, C. Lo Iacono, D. Casalbore, J. S. Davies, I. Kane, V. A. I. Huvenne, P. T. Harris, Transport and accumulation of litter in submarine canyons: A geoscience perspective. *Front. Mar. Sci.* **10**, 1–22 (2023).

2. M. L. Baker, S. Hage, P. J. Talling, S. Acikalin, R. G. Hilton, N. Haghipour, S. C. Ruffell, E. L. Pope, R. S. Jacinto, M. A. Clare, S. Sahin, Globally significant mass of terrestrial organic carbon efficiently transported by canyon-flushing turbidity currents. *Geology* **52**, 631–636 (2024).

3. W. R. Normark, P. R. Carlson, Giant submarine canyons: Is size any clue to their importance in the rock record? *Geol. Soc. Am. Spec. Paper* **370**, 1–15 (2003).

4. P. T. Harris, M. Macmillan-Lawler, J. Rupp, E. K. Baker, Geomorphology of the oceans. *Mar. Geol.* **352**, 4–24 (2014).

5. J. A. Covault, S. A. Graham, Submarine fans at all sea-level stands: Tectono-morphologic and climatic controls on terrigenous sediment delivery to the deep sea. *Geology* **38**, 939–942 (2010).

6. W. L. Fisher, W. E. Galloway, R. J. Steel, C. Olariu, C. Kerans, D. Mohrig, Deep-water depositional systems supplied by shelf-incising submarine canyons: Recognition and significance in the geologic record. *Earth Sci. Rev.* **214**, 103531 (2021).

7. P. J. Talling, S. Hage, M. L. Baker, T. S. Bianchi, R. G. Hilton, K. L. Maier, The global turbidity current pump and its implications for organic carbon cycling. *Ann. Rev. Mar. Sci.* **16**, 105–133 (2024).

8. Z. Huang, S. L. Nichol, P. T. Harris, M. J. Caley, Classification of submarine canyons of the Australian continental margin. *Mar. Geol.* **357**, 362–383 (2014).

9. Z. Huang, T. A. Schlacher, S. Nichol, A. Williams, F. Althaus, R. Kloser, A conceptual surrogacy framework to evaluate the habitat potential of submarine canyons. *Prog. Oceanogr.* **169**, 199–213 (2018).

10. P. T. Harris, T. Whiteway, Global distribution of large submarine canyons: Geomorphic differences between active and passive continental margins. *Mar. Geol.* **285**, 69–86 (2011).

11. Z. R. Jobe, D. R. Lowe, S. J. Uchytil, Two fundamentally different types of submarine canyons along the continental margin of Equatorial Guinea. *Mar. Pet. Geol.* **28**, 843–860 (2011).

12. A. Bernhardt, W. Schwanghart, Where and why do submarine canyons remain connected to the shore during sea-level rise? Insights from global topographic analysis and Bayesian regression. *Geophys. Res. Lett.* **48**, 1–15 (2021).

13. M. S. Heijnen, F. Mienis, A. R. Gates, B. J. Bett, R. A. Hall, J. Hunt, I. A. Kane, C. Pebody, V. A. I. Huvenne, E. L. Soutter, M. A. Clare, Challenging the highstand-dormant paradigm for land-detached submarine canyons. *Nat. Commun.* **13**, 1–11 (2022).

14. L. Pratson, B. Coakley, A model for the headward erosion of submarine canyons induced by downslope-eroding sediment flows. *Geol. Soc. Am. Bull.* **108**, 225–234 (1996).

15. L. F. Pratson, W. B. F. Ryan, G. S. Mountain, D. C. Twichell, Submarine canyon initiation by downslope-eroding sediment flows: Evidence in late Cenozoic strata on the New Jersey continental slope. *Geol. Soc. Am. Bull.* **106**, 395–412 (1994).

16. S. Y. J. Lai, T. P. Gerber, D. Amblas, An experimental approach to submarine canyon evolution. *Geophys. Res. Lett.* **43**, 2741–2747 (2016).

17. M. E. Smith, N. J. Finnegan, E. R. Mueller, R. J. Best, Durable terrestrial bedrock predicts submarine canyon formation. *Geophys. Res. Lett.* **44**, 10,332–10,340 (2017).

18. M. S. Heijnen, M. A. Clare, M. J. B. Cartigny, P. J. Talling, S. Hage, D. G. Lintern, C. Stacey, D. R. Parsons, S. M. Simmons, Y. Chen, E. J. Sumner, J. K. Dix, J. E. Hughes Clarke, Rapidly-migrating and internally-generated knickpoints can control submarine channel evolution. *Nat. Commun.* **11**, 3129 (2020).

19. D. C. Twichell, D. G. Roberts, Morphology, distribution, and development of submarine canyons on the United States Atlantic continental slope between Hudson and Baltimore Canyons. *Geology* **10**, 408–412 (1982).

20. J. A. Farre, B. A. McGregor, W. B. F. Ryan, J. M. Robb, Breaching the shelfbreak: Passage from youthful to mature phase in submarine canyon evolution. *SEPM Spec. Publ.* **33**, 25–39 (1983).

21. L. Wan, S. Hurter, V. Bianchi, T. Salles, Z. Zhang, X. Yuan, Combining stratigraphic forward modeling and susceptibility mapping to investigate the origin and evolution of submarine canyons. *Geomorphology* **398**, 108047 (2022).

22. C. Petit, S. Migeon, M. Coste, Numerical models of continental and submarine erosion: Application to the northern Ligurian margin (Southern Alps, France/Italy). *Earth Surf. Process. Landf.* **40**, 681–695 (2015).

23. M. E. Smith, S. H. Werner, D. Buscombe, N. J. Finnegan, E. J. Sumner, E. R. Mueller, Seeking the shore: Evidence for active submarine canyon head incision due to coarse sediment supply and focusing of wave energy. *Geophys. Res. Lett.* **45**, 12,403–12,413 (2018).

24. A. Baddeley, E. Rubak, R. Turner, *Spatial Point Patterns: Methodology and Applications with R* (Apple Academic Press Inc. Boca Raton, 2015).

25. A. Baddeley, G. Nair, S. Rakshit, G. McSwiggan, T. M. Davies, Analysing point patterns on networks — A review. *Spat. Stat.* **42**, 100435 (2021).

26. W. Schwanghart, C. Molkenthin, D. Scherler, A systematic approach and software for the analysis of point patterns on river networks. *Earth Surf. Process. Landf.* **46**, 1847–1862 (2021).

27. J. J. Becker, D. T. Sandwell, W. H. F. Smith, J. Braud, B. Binder, J. Depner, D. Fabre, J. Factor, S. Ingalls, S. H. Kim, R. Ladner, K. Marks, S. Nelson, A. Pharaoh, R. Trimmer, J. von Rosenberg, G. Wallace, P. Weatherall, Global bathymetry and elevation data at 30 arc seconds resolution: SRTM30_PLUS. *Mar. Geod.* **32**, 355–371 (2009).

28. B. Lehner, K. Verdin, A. Jarvis, New global hydrography derived from spaceborne elevation data. *Eos Trans. Am. Geophys. Union* **89**, 93–94 (2008).

29. E. Luijendijk, T. Gleeson, N. Moosdorf, Fresh groundwater discharge insignificant for the world's oceans but important for coastal ecosystems. *Nat. Commun.* **11**, 1260 (2020).

30. E. O. Straume, C. Gaina, S. Medvedev, K. Hochmuth, K. Gohl, J. M. Whittaker, R. Abdul Fattah, J. C. Doornenbal, J. R. Hopper, GlobSed: Updated total sediment thickness in the world's oceans. *Geochem. Geophys. Geosyst.* **20**, 1756–1772 (2019).

31. M. Seton, R. D. Müller, S. Zahirovic, S. Williams, N. M. Wright, J. Cannon, J. M. Whittaker, K. J. Matthews, R. McGirr, A global data set of present-day oceanic crustal age and seafloor spreading parameters. *Geochem. Geophys. Geosyst.* **21**, 1–15 (2020).

32. B. M. Fekete, C. J. Vörösmarty, W. Grabs, High-resolution fields of global runoff combining observed river discharge and simulated water balances. *Glob. Biogeochem. Cycles* **16**, 15-1–15-10 (2002).

33. J. P. M. Syvitski, A. Kettner, Sediment flux and the Anthropocene. *Phil. Trans. R. Soc. A* **369**, 957–975 (2011).

34. K. Shedlock, D. Giardini, G. Gruenthal, P. Zhang, The GSHAP global seismic hazard map. *Seismol. Res. Lett.* **71**, 679–686 (2003).

35. N. Moosdorf, S. Cohen, C. von Hagke, A global erodibility index to represent sediment production potential of different rock types. *Appl. Geogr.* **101**, 36–44 (2018).

36. N. Boers, B. Bookhagen, H. M. J. Barbosa, N. Marwan, J. Kurths, J. A. Marengo, Prediction of extreme floods in the eastern Central Andes based on a complex networks approach. *Nat. Commun.* **5**, 1–7 (2014).

37. A. Bernhardt, D. Melnick, J. Jara-Muñoz, B. Argandoña, J. González, M. R. Strecker, Controls on submarine canyon activity during sea-level highstands: The Biobío Canyon system offshore Chile. *Geosphere* **11**, 1226–1255 (2015).

38. P. Weatherall, K. M. Marks, M. Jakobsson, T. Schmitt, S. Tani, J. E. Arndt, M. Rovere, D. Chayes, V. Ferrini, R. Wigley, A new digital bathymetric model of the world's oceans. *Earth Space Sci.* **2**, 331–345 (2015).

39. L. Anselin, X. Li, Operational local join count statistics for cluster detection. *J. Geogr. Syst.* **21**, 189–210 (2019).

40. D. L. Orange, R. S. Anderson, N. A. Breen, Regular canyon spacing in the submarine environment: The link between hydrology and geomorphology. *GSA Today* **4**, 35–39 (1994).

41. L. H. Bührig, L. Colombera, M. Patacci, N. P. Mountney, W. D. McCaffrey, A global analysis of controls on submarine-canyon geomorphology. *Earth Sci. Rev.* **233**, 104150 (2022).

42. D. B. O'Grady, J. P. M. Syvitski, "Predicting profile geometry of continental slopes with a multiprocess sedimentation model," in *Geologic Modeling and Simulation - Sedimentary Systems*, D. F. Merriam, J. C. Davis, Eds. (Kluwer Academic/Plenum Publishers, New York, 2001), pp. 99–117.

43. L. F. Pratson, C. A. Nittrouer, P. L. Wiberg, M. S. Steckler, J. B. Swenson, D. A. Cacchione, J. A. Karson, A. B. Murray, M. A. Wolinsky, T. P. Gerber, B. L. Mullenbach, G. A. Spinelli, C. S.Fulthorpe, D. B. O'Grady, G. Parker, N. W. Driscoll, R. L. Burger, C. Paola, D. L. Orange, M. E. Field, C. T. Friedrichs, J. J. Fedele, "Seascape evolution on clastic continental shelves and slopes," in *Continental Margin Sedimentation: From Sediment Transport to Sequence Stratigraphy*, C. A. Nittrouer, J. A. Austin, M. E. Field, J. H. Kravitz, J. P.M. Syvitski, P. L. Wiberg, Eds. (International Association of Sedimentologists, 2007), pp. 339–380.

44. I. Anell, The fourth slope: A fundamental new classification of continental margins. *Basin Res.* **36**, e12863 (2024).

45. E. W. Adams, J. A. Kenter, "So different, yet so similar: Comparing and contrasting siliciclastic and carbonate slopes," in *Deposits, Architecture and Controls of Carbonate Margin, Slope, Basinal Settings*, K. Verwer, T. E. Playton, P. M. Harris, Eds. (SEPM Special Publications, 2014), p. 105.

46. B. G. McAdoo, L. F. Pratson, D. L. Orange, Submarine landslide geomorphology, US continental slope. *Mar. Geol.* **169**, 103–136 (2000).

47. R. Harders, C. R. Ranero, W. Weinrebe, J. H. Behrmann, Submarine slope failures along the convergent continental margin of the Middle America Trench. *Geochem. Geophys. Geosyst.* **12**, Q05S32 (2011).

48. U. S. ten Brink, B. D. Andrews, N. C. Miller, Seismicity and sedimentation rate effects on submarine slope stability. *Geology* **44**, 563–566 (2016).

49. A. Bernhardt, W. Schwanghart, "Global dataset of submarine canyon heads combined with terrestrial and marine topographic and oceanographic parameters," GFZ Data Serv, 2021; https://doi.org/10.5880/fidgeo.2021.008.

50. K. G. Miller, M. A. Kominz, J. V. Browning, J. D. Wright, G. S. Mountain, M. E. Katz, P. J. Sugarman, B. S. Cramer, N. Christie-Blick, S. F. Pekar, The phanerozoic record of global sea-level change. *Science* **310**, 1293–1298 (2005).

51. C. K. Harris, P. Wiberg, Across-shelf sediment transport: Interactions between suspended sediment and bed sediment. *J. Geophys. Res. Ocean* **107**, 8-1–8-12 (2002).

52. L. Lombardo, T. Opitz, R. Huser, Point process-based modeling of multiple debris flow landslides using INLA: An application to the 2009 Messina disaster. *Stoch. Environ. Res. Risk Assess.* **32**, 2179–2198 (2018).

53. C. Palmiotto, M. F. Loreto, Regional scale morphological pattern of the Tyrrhenian Sea: New insights from EMODnet bathymetry. *Geomorphology* **332**, 88–99 (2019).

54. N. M. Cagatay, G. Ucarkus, K. K. Eris, P. Henry, L. Gasperini, A. Polonia, "Submarine canyons of the Sea of Marmara," in *Submar. Canyon Dyn. Mediterr. Tribut. Seas, CIESM Work. Monogr. no. 47* (CIESM: The Mediterranean Science Commission, 2015), pp. 123–135.

55. J. Bourget, S. Zaragosi, N. Ellouz-Zimmermann, N. Mouchot, T. Garlan, J.-L. Schneider, V. Lanfumey, S. Lallemant, Turbidite system architecture and sedimentary processes along

topographically complex slopes: The Makran convergent margin. *Sedimentology* **58**, 376–406 (2011).

56. R. M. Pratt, B. C. Heezen, Topography of the Blake Plateau. *Deep Sea Res. Oceanogr. Abstr.* **11**, 721–728 (1964).

57. V. V. Ivanov, G. I. Shapiro, J. M. Huthnance, D. L. Aleynik, P. N. Golovin, Cascades of dense water around the world ocean. *Prog. Oceanogr.* **60**, 47–98 (2004).

58. A. L. De Geest, B. L. Mullenbach, P. Puig, C. A. Nittrouer, T. M. Drexler, X. Durrieu de Madron, D. L. Orange, Sediment accumulation in the western Gulf of Lions, France: The role of Cap de Creus Canyon in linking shelf and slope sediment dispersal systems. *Cont. Shelf Res.* **28**, 2031–2047 (2008).

59. T. Mahjabin, C. Pattiaratchi, Y. Hetzel, Occurrence and seasonal variability of dense shelf water cascades along Australian continental shelves. *Sci. Rep.* **10**, 11–14 (2020).

60. D. L. Orange, N. A. Breen, The effects of fluid escape on accretionary wedges seepage force, slope failure, headless submarine canyons, and vents. *J. Geophys. Res.* **97**, 9277–9295 (1992).

61. F. Herman, J. D. Champagnac, Plio-Pleistocene increase of erosion rates in mountain belts in response to climate change. *Terra Nov.* **28**, 2–10 (2016).

62. P. J. Paris, J. P. Walsh, D. R. Corbett, Where the continent ends. *Geophys. Res. Lett.* **43**, 12208–12216 (2016).

63. J. Baztan, S. Berné, J.-L. Olivet, M. Rabineau, D. Aslanian, M. Gaudin, J.-P. Réhault, M. Canals, Axial incision: The key to understand submarine canyon evolution (in the western Gulf of Lion). *Mar. Pet. Geol.* **22**, 805–826 (2005).

64. J. K. Mitchell, G. R. Holdgate, M. W. Wallace, Pliocene - Pleistocene history of the Gippsland Basin outer shelf and canyon heads, southeast Australia. *Aust. J. Earth Sci.* **54**, 49–64 (2007).

65. A. Antobreh, S. Krastel, Morphology, seismic characteristics and development of Cap Timiris Canyon, offshore Mauritania: A newly discovered canyon preserved-off a major arid climatic region. *Mar. Pet. Geol.* **23**, 37–59 (2006).

66. V. Regard, R. Almar, M. Graffin, E. Anthony, R. Ranasighe, S. Carretier, P. Maffre, The contribution of diminishing river sand loads to beach erosion worldwide. *Nat. Hazards Earth Syst. Sci.*, 10.5194/nhess-2023-165 (2023).

67. T. Salles, L. Husson, P. Rey, C. Mallard, S. Zahirovic, B. H. Boggiani, N. Coltice, M. Arnould, Hundred million years of landscape dynamics from catchment to global scale. *Science* **379**, 918–923 (2023).

68. R. G. Hilton, A. J. West, Mountains, erosion and the carbon cycle. *Nat. Rev. Earth Environ.* **1**, 284–299 (2020).

69. T. Whiteway, Australian bathymetry and topography grid, June 2009 (Geoscience Australia, Canberra, 2009).

70. W. Schwanghart, D. Scherler, Short Communication: TopoToolbox 2 - MATLAB-based software for topographic analysis and modeling in Earth surface sciences. *Earth Surf. Dyn.* **2**, 1–7 (2014).

71. S. Muis, M. Verlaan, H. C. Winsemius, J. C. J. H. Aerts, P. J. Ward, A global reanalysis of storm surges and extreme sea levels. *Nat. Commun.* **7**, 11969 (2016).

72. D. Zamrsky, G. H. P. O. Essink, E. H. Sutanudjaja, L. P. H. R. Van Beek, M. F. P. Bierkens, Offshore fresh groundwater in coastal unconsolidated sediment systems as a potential fresh water source in the 21st century. *Environ. Res. Lett.* **17**, 014021 (2022).

73. L. Hoeltgen, A. Kleefeld, I. Harris, M. Breuss, Theoretical foundation of the weighted laplace inpainting problem. *Appl. Math.* **64**, 281–300 (2019).

74. G. J. Weltje, R. Tjallingii, Calibration of XRF core scanners for quantitative geochemical logging of sediment cores: Theory and application. *Earth Planet. Sci. Lett.* **274**, 423–438 (2008).

75. R. Tolosana-Delgado, Uses and misuses of compositional data in sedimentology. *Sediment. Geol.* **280**, 60–79 (2012).

76. E. Makalic, D. F. Schmidt, High-dimensional Bayesian regularised regression with the bayesreg package. arXiv:1611.06649 [stat.CO] (2016).

77. E. Makalic, D. F. Schmidt, "A simple Bayesian algorithm for feature ranking in high dimensional regression problems," in *AI 2011: Advances in Artificial Intelligence*, *Lecture Notes in Computer Science*, D. Wang, M. Reynolds, Eds. (Springer, 2011), vol. 7106, pp. 223–230.

78. J. Verhaegen, G. J. Weltje, D. Munsterman, Workflow for analysis of compositional data in sedimentary petrology: Provenance changes in sedimentary basins from spatio-temporal variation in heavy-mineral assemblages. *Geol. Mag.* **156**, 1111–1130 (2019).

79. K. G. van den Boogaart, R. Tolosana-Delgado, *Analyzing Compositional Data with R* (Springer, 2011).

80. O. Korup, Bayesian geomorphology. *Earth Surf. Process. Landf.* **46**, 151–172 (2021).

81. R. Tibshirani, Regression shrinkage and selection via the lasso: A retrospective. *J. R. Stat. Soc. Ser. B Stat. Methodol.* **73**, 273–282 (2011).

82. S. Watanabe, Asymptotic equivalence of Bayes cross validation and widely applicable information criterion in singular learning theory. *J. Mach. Learn. Res.* **11**, 3571–3594 (2010).

83. A. Bernhardt, W. Schwanghart, Submarine canyon distribution and environmental controls: A global dataset for point pattern analysis, GFZ Data Services, 2025; https://doi.org/10.5880/fidgeo.2025.031.

84. W. B. F. Ryan, S. M. Carbotte, J. O. Coplan, S. O'Hara, A. Melkonian, R. Arko, R. A. Weissel, V. Ferrini, A. Goodwillie, F. Nitsche, J. Bonczkowski, R. Zemsky, Global multi-resolution topography synthesis. *Geochem. Geophys. Geosyst.* **10**, Q03014 (2009).
